# DNA methylation alterations at RE1-silencing transcription factor binding sites and their flanking regions in cancer

**DOI:** 10.1186/s13148-023-01514-9

**Published:** 2023-06-10

**Authors:** Ana Florencia Vega-Benedetti, Eleonora Loi, Loredana Moi, Patrizia Zavattari

**Affiliations:** Department of Biomedical Sciences, Unit of Biology and Genetics, University of Cagliari, 09042 Cagliari, Italy

**Keywords:** DNA methylation alterations, RE1-silencing transcription factor, REST, Brain cancer, Gastrointestinal cancer, Blood cancer

## Abstract

**Background:**

DNA methylation changes, frequent early events in cancer, can modulate the binding of transcription factors. RE1-silencing transcription factor (REST) plays a fundamental role in regulating the expression of neuronal genes, and in particular their silencing in non-neuronal tissues, by inducing chromatin modifications, including DNA methylation changes, not only in the proximity of its binding sites but also in the flanking regions. REST has been found aberrantly expressed in brain cancer and other cancer types. In this work, we investigated DNA methylation alterations at REST binding sites and their flanking regions in a brain cancer (pilocytic astrocytoma), two gastrointestinal tumours (colorectal cancer and biliary tract cancer) and a blood cancer (chronic lymphocytic leukemia).

**Results:**

Differential methylation analyses focused on REST binding sites and their flanking regions were conducted between tumour and normal samples from our experimental datasets analysed by Illumina microarrays and the identified alterations were validated using publicly available datasets. We discovered distinct DNA methylation patterns between pilocytic astrocytoma and the other cancer types in agreement with the opposite oncogenic and tumour suppressive role of REST in glioma and non-brain tumours.

**Conclusions:**

Our results suggest that these DNA methylation alterations in cancer may be associated with REST dysfunction opening the enthusiastic possibility to develop novel therapeutic interventions based on the modulation of this master regulator in order to restore the aberrant methylation of its target regions into a normal status.

**Supplementary Information:**

The online version contains supplementary material available at 10.1186/s13148-023-01514-9.

## Background

Nowadays, it is recognized that DNA methylation has a key role in different biological processes including gene regulation, nucleosome positioning and binding of transcription factors. DNA methylation mainly occurs in the context of CpG dinucleotides, enriched in regions defined as CpG islands (CGIs), frequently located in gene promoters. It is known that methylation alterations are early events in cancer and thus they could also be involved in tumour onset and progression [1, 2]. In fact, promoter hypermethylation is often associated with specific gene downregulation in tumours [1, 3]. In this context, given that cytosine methylation modifies DNA structure, it can affect the binding of transcription factors (TFs) resulting in gene expression dysregulation. It is well accepted that hypermethylation can avoid TFs binding. Indeed, methylation within CTCF binding sites reduces its DNA binding ability, a key mechanism of expression regulation in clustered-*PCDH* genes [4]. Moreover, methylation adjacent to transcription factor binding sites such as activator protein 1 (AP-1) and Specificity protein (Sp) Sp1/Sp3 sites negatively affects their binding, leading to changes in transcription modulation [5, 6]. However, not all TFs are influenced in the same way by methylation; some of them require methylated DNA sequences to efficiently bind, while others bind to their motifs despite DNA methylation status [7]. Recently, it has been developed the Methmotif database that integrates TF binding sites with DNA methylation profile in different cell lines [8]. As highlighted in these works DNA methylation associated with TF motifs plays a relevant role for their function. In particular, RE1-silencing transcription factor (REST), alias neuron-restrictive silencer factor (NRSF), modulates the transcription of neuronal genes. REST protein structure consists of a DNA binding domain that includes eight highly conserved zinc fingers, and two repressor domains located in the amino and carboxy terminal regions [9–11]. REST recognizes its binding motifs, known as neuron-restrictive silencer element (NRSE) or repressor element 1 (RE1). In general, these regulatory elements are near the transcription start sites (TSS) of non-coding RNA genes and protein-coding genes that must be expressed in neurons but not in other cell types [12, 13]. These target genes encode proteins that are involved in excitability, neurotransmitter release, and control of transmembrane potential which are essential mechanisms for the nervous system but harmful for non-neuronal cells. Therefore, REST has a key role in silencing these genes in cells outside the nervous system [13]. Gene expression regulation orchestrated by REST is a complex mechanism that includes a great variety of cofactors. In non-neuronal cell types, where REST is ubiquitously expressed, it binds to its conserved motif and recruits two corepressors: mammalian SIN3 transcription regulator family member A (mSin3a) and REST corepressor 1 (CoREST). Furthermore, this complex mediates the recruitment of additional chromatin modulators: HDACs, MeCP2, H3K9 methyltransferases G9a and SUV39H1, the H3K4 demethylase LSD1, and the DNA methyltransferase DNMT1, leading to chromatin compaction and permanent gene silencing. REST complex differently regulates its target genes during development. In fact, in multipotent embryonic stem cells and in neuronal progenitors REST-target genes are transcribed but at low levels and in mostly differentiated neurons REST is downregulated, thus reducing the presence of REST inhibitor complexes at chromatin and allowing active transcription of neuronal genes [9–11]. Furthermore, during neuronal differentiation REST may also play the role of activator through interaction with a small double stranded non-coding RNA [10]. Of note, REST-dependent chromatin modification could affect not only the immediate region around NRSE motif but also a chromosomal region that flank a NRSE sequence [13, 14]. Stadler and collaborators demonstrated that the inhibition of REST in embryonic stem cells is associated with an increased DNA methylation in its target regions, while the re-expression of REST in a knockout model restores the un-methylated state [15]. The exact mechanism of how REST modulates DNA methylation at NRSEs and neighbouring regions is not clear, however there may be an interplay between REST and the DNA methylation machinery [16]. In fact, in REST knockout embryonic stem cells the absence of REST correlates with an increase of DNA methylation at NRSE, supporting the hypothesis that this TF modulates methylation status of its regulatory sequences [17]. Moreover, REST may autoregulate its own expression through NRSE located in its promoter and it is also regulated by non-coding RNAs [12].

The important role of REST regulation system in the remodeling of the cellular epigenome has been investigated in different contexts. Particularly, this mechanism has been studied in the regulation of clustered protocadherin (*PCDH*) gene expression. In fact, the enhancer element HS5-1 of *PCDH* cluster α also contains a REST binding motif. Furthermore, it has been demonstrated a strong REST binding to the RE1 sequence within the HS5-1 element in non-neuronal human cell lines, leading to gene silencing [18, 19]. This RE1 sequence can modulate the transcription of multiple *PCDH* genes included in the cluster α, providing further evidence that these sequences could act as long-range regulators [18]. REST regulation mechanism is also involved in mouse heart development, presenting a coordinated activity of REST and DNMT3B [20]. Indeed, Zhang et al. (2017) reported that non-CpG methylation depends on DNMT3B and plays a more dynamic role respect to CpG methylation in NRSEs in developing hearts [20]. Regarding another context, the role of REST regulation in cancer is not fully elucidated. Recently, in U87 glioma cell line, it has been reported a CpG methylation alteration that overlaps with a REST motif, affecting the interaction between this TF with its binding site [21]. Increased REST expression was reported to promote tumour progression in glioma, suggesting its role as an oncogene in brain cancer [22]. Moreover, in medulloblastomas REST expression is increased and it is associated with *Ptch1* repression, *Pten* decrease and activation of AKT [23]. On the other hand, in non-brain tumours REST plays a tumour suppressor role. In fact, in other cancer types such as breast cancer, *REST* loss of function and the presence of *REST4* transcript variant correlate with target gene dysregulation and tumour aggressiveness [24, 25], whereas in colorectal cancer *REST* is frequently deleted and this is associated with malignancy transformation [26]. In prostate and small cell lung cancer gene dysregulation mediated by REST and the expression of REST variants are associated with a neuroendocrine phenotype [27–29].

**Fig. 1:**
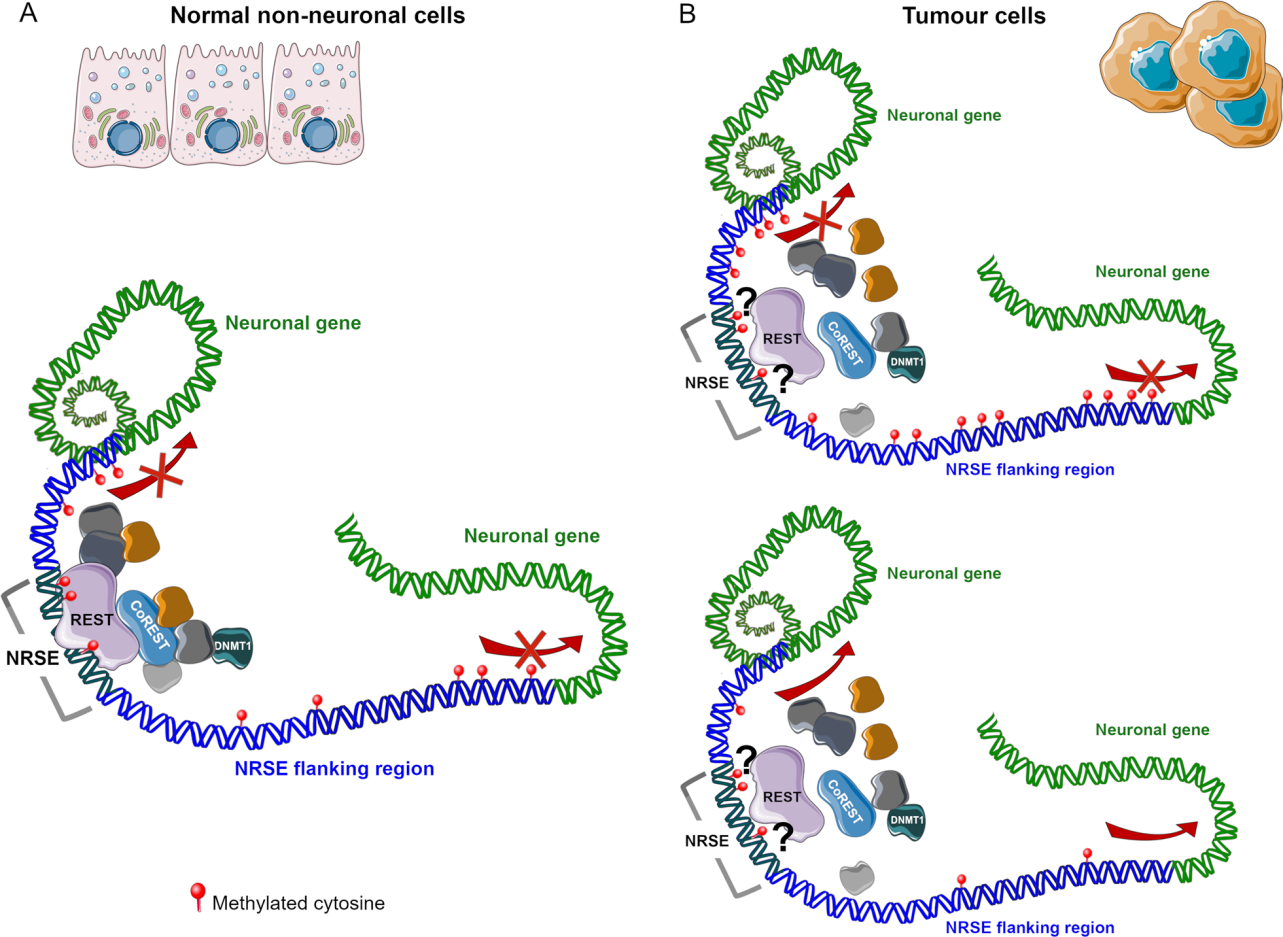
Illustration of the role of REST in the regulation of neuronal genes in non-neuronal normal cells (**A**) and two possible scenario of DNA methylation pattern and REST dysregulation in tumour cells leading to different neuronal gene expression modulation (**B**)

Given the opposite role of REST in normal neuronal and non-neuronal tissues, its involvement in several cancers, the implication of DNA methylation alterations in tumorigenesis and the probable interplay between REST and the DNA methylation machinery, we wonder whether the DNA methylation pattern at NRSEs also differ between tumour types. Interestingly, our previous studies have shown that neuronal genes are frequently epigenetically dysregulated in non-brain cancers [1, 30–34], also supported by other evidence [35]. Since REST plays a crucial role in the regulation of neuronal-related genes, we decided to analyse the DNA methylation pattern at NRSE in the previously analysed tumours. To the best of our knowledge, the current work is the first one to investigate the methylation status of CpGs at NRSEs and their neighbouring regions in colorectal and biliary tract cancers (CRC and BTC, respectively), pilocytic astrocytoma (PA), and chronic lymphocytic leukemia (CLL). Our results demonstrate that REST binding sites and their flanking regions are frequently altered in their methylation status in tumours, showing distinct patterns between PA and the other analysed cancers suggesting that REST-mediated regulation mechanism is also different during malignancy between neuronal and non-neuronal tissues as shown in the exemplificative Fig. 1.

## Results

### Aberrantly methylated NRSEs in cancer

Differential methylation analyses focused on REST binding sites were carried out between tumour and normal samples from the experimental datasets and the identified alterations were validated using publicly available datasets (Additional file 1: Fig. S1). Aberrant DNA methylation at NRSEs was detected in all the analysed cancers, PA, CRC, BTC and CLL. However, interesting differences were observed between tumours. Very different DNA methylation alteration patterns were observed among PA, gastrointestinal tumours and CLL. The analysis showed altered NRSEs associated with the following genes involved in neuronal processes: *PCDHG* cluster, *BARHL2*, *OTX2*, *NXPH1* and *RARA*. Two others selected NRSEs are associated with *LOC93429*, a long non-coding RNA, and the pseudogene *HCG22*. Interestingly, these NRSE presented opposite DNA methylation pattern between PA and gastrointestinal tumours, whereas no alteration in these NRSE regions was detected in CLL, suggesting a different regulation mechanism in tumour types.

### NRSE methylation alterations in pilocytic astrocytoma

**Fig. 2:**
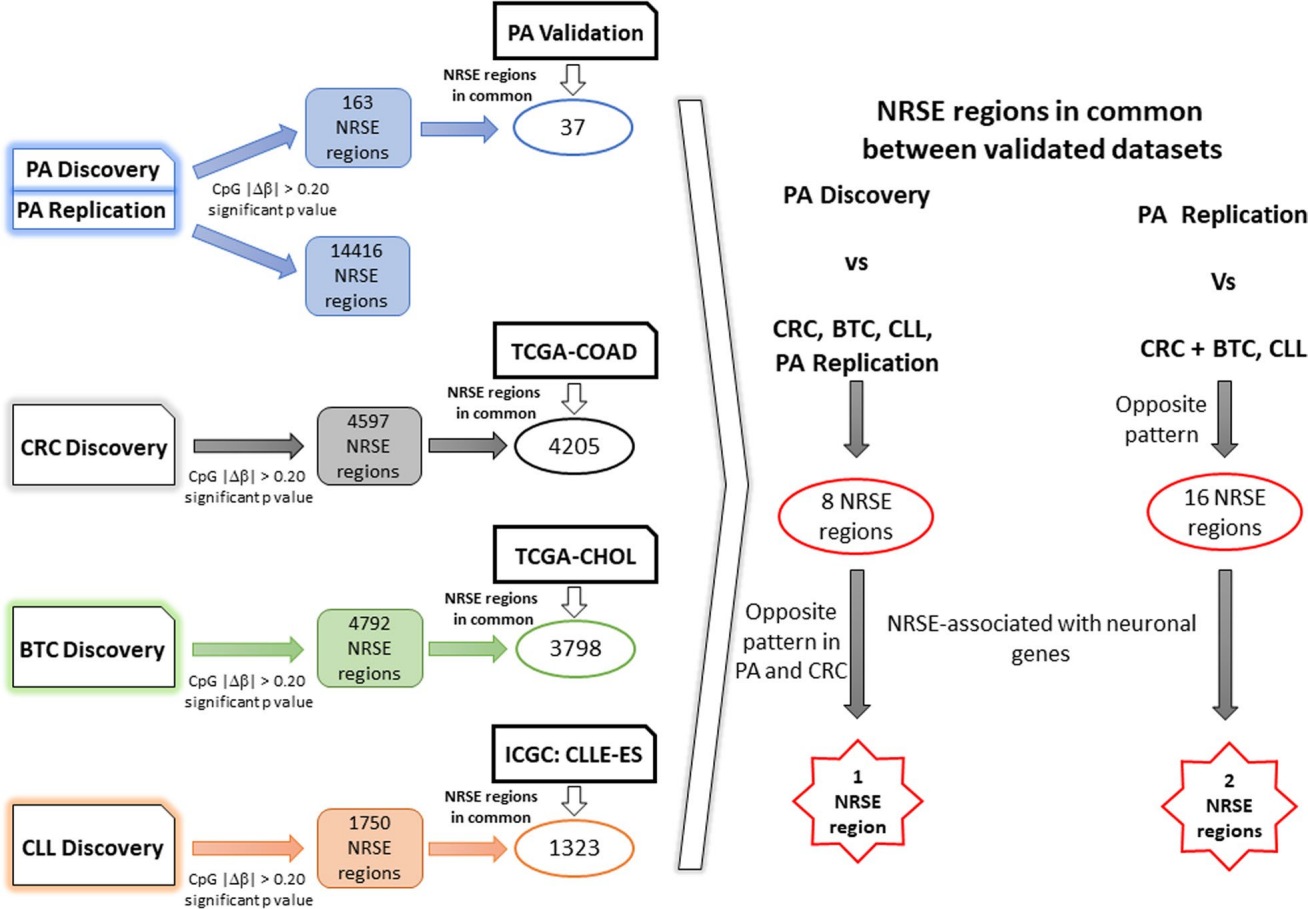
Analysis workflow. Workflow of DNA methylation alterations selection: from a genome-wide to an NRSE approach

We investigated the methylation status of CpG sites within REST binding sites and in their flanking regions considering a distance of 2 kb (see “Methods” Section), collectively defined as NRSE region in our paper from now onwards, in 20 PA and four normal samples (PA Discovery dataset). The differential methylation analysis showed 120 altered CpGs in 163 NRSE regions (Fig. 2). We detected 80 hypomethylated and 40 hypermethylated CpG sites. Most of the altered CpGs, 54, are positioned in open sea, 46 in CGI flanking regions and 20 within CGIs.

**Fig. 3:**
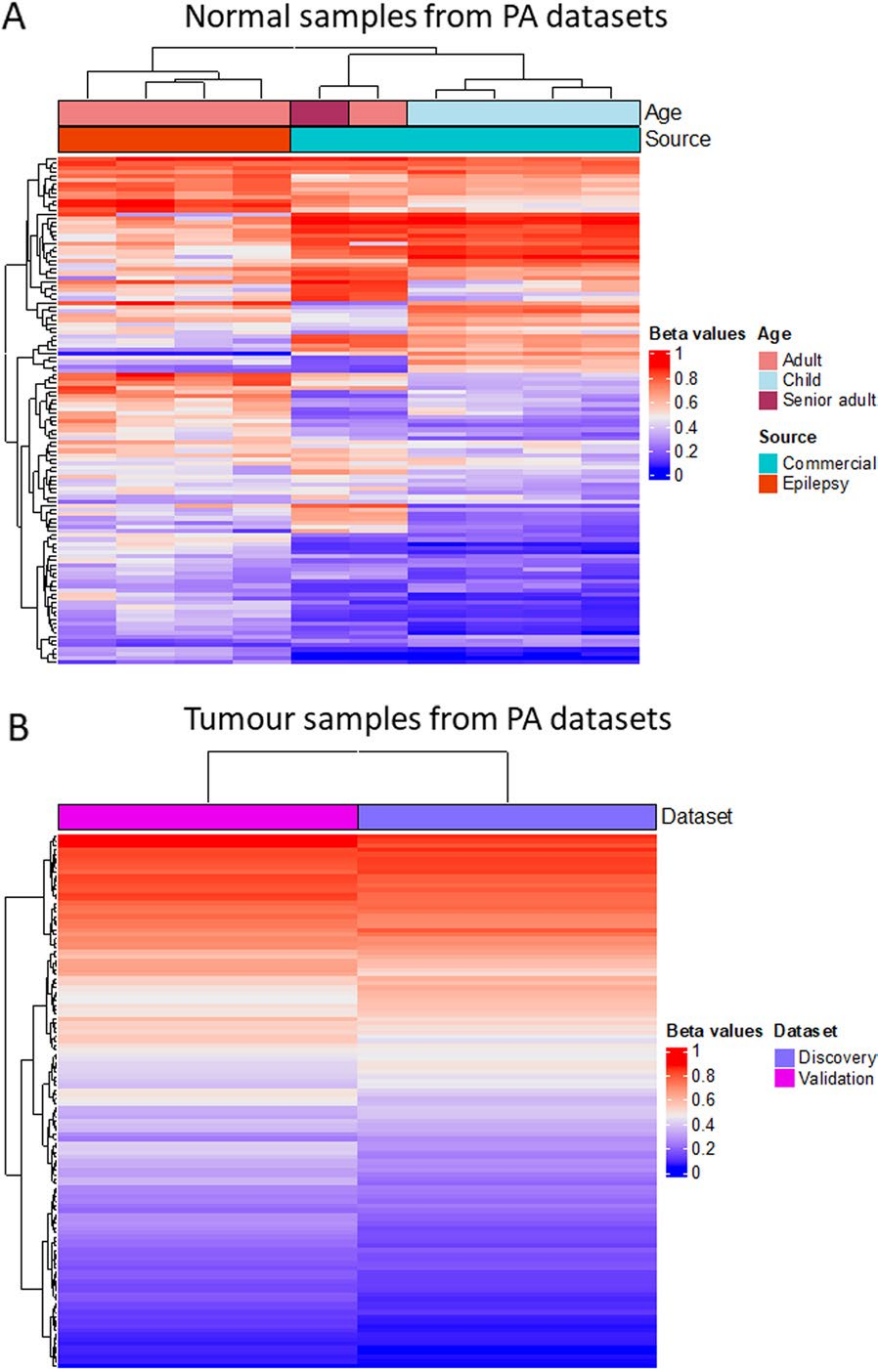
Heatmaps of the 120 altered CpG sites in PA Discovery and Validation datasets. Unsupervised hierarchical clustering analysis based on the *β* value of each control sample from both datasets (**A**) and on the average *β* value of tumour samples from both datasets (**B**)

Few of these alterations, 26 CpG sites, associated with 37 NRSE regions, were successfully cross-validated in the PA Validation dataset (Fig. 2). The contradictory results may reside in the different methylation values between the normal samples included in each dataset, considering both the different source and age (Fig. 3A).

#### NRSE methylation pattern among normal brain samples

It should be considered that the origin of normal samples is different between the two datasets, i.e. from epileptic subjects in PA Discovery and commercial sources in PA Validation. For this reason, we further checked the methylation values of the altered CpGs among normal brain samples (epilepsy vs non-epilepsy) and their association with known epilepsy-related genes. Some CpGs were differently methylated between epileptic and non-epileptic subjects. One altered CpG site (cg10708793) overlaps with an NRSE region, NRSE at chr17:40,264,438–40,264,968, associated with an enhancer and *DHX58* gene. It displays high methylation value (*β* value = 0.66) in control samples from the PA Validation dataset and this methylation level is observed in almost all CpGs of the NRSE region, whereas the same CpG, the only one interrogated in PA Discovery, presents low *β* value (*β* value = 0.06) in epileptic samples. The differential methylation analysis between epileptic and control samples results in a hypomethylation event with a Δ*β* of − 0.6. Other two interrogated CpGs, cg20289949 and cg01561916, located at NRSE region, NRSE at chr2:43,019,500–43,020,030, have higher β methylation values (0.35 and 0.41) in the epileptic samples than in the control samples (0.10 and 0.14) from the PA Validation. The differential methylation values for these CpG sites are − 0.25 and − 0.27, respectively. This altered NRSE region overlaps with a CGI and is associated with *HAAO* gene.

Moreover, the different age observed not only between controls from the discovery and the validation dataset but also within the validation dataset is another factor to consider (Fig. 3A). To note, our discovery dataset included four adult control samples while the validation dataset consisted of six control samples from four children below 10 months of birth and two subjects whose age were 33 (Adult) and 87 (Senior adult) years old. UHC showed a clear distinction between controls that are divided in three clusters, children controls, epileptic adult patients, and non-epileptic adult samples (Fig. 3A), whereas tumours present similar mean *β* values in both datasets (Fig. 3B). The age of PA patients is more homogeneous in both discovery and validation datasets. In fact, tumours did not separate in branches according to age (Additional file 1: Fig. S2).

Given the mean age-group of tumour samples is 8 years old, we considered correct to compare them with the children controls defining the PA Replication dataset that will be used as an independent cohort. In this dataset we identified 15,656 altered CpG sites (9508 hypermethylated and 6148 hypomethylated) associated with 14,416 NRSE regions (Fig. 2). We detected 2160 altered CpGs in CGI, 6490 in CGI flanking regions and 7006 in open sea.

### NRSE methylation alterations in colorectal cancer

The methylation analysis conducted on 18 CRC and four normal samples (CRC Discovery dataset) showed 5845 differentially methylated CpG sites (3059 hypermethylated and 2786 hypomethylated) associated with 4597 NRSE regions (Fig. 2). Most of these CpGs, 2466, were located within CGI, 1432 in CGI flanking regions and 1947 in open sea.

We successfully cross-validated 5359 CpG alterations within 4205 NRSE regions in TCGA-COAD dataset (Fig. 2).

The list of genes whose associated NRSEs were significantly altered, was subjected to gene enrichment analysis by DAVID. The most affected pathways by hypermethylation events were: cell adhesion molecules, neuroactive ligand-receptor interaction, GABAergic synapse, cAMP and calcium signalling pathway, cholinergic and glutamatergic synapse, among others (Additional file 1: Fig. S3A). Genes associated with hypermethylated CpG sites mainly encode developmental and DNA-binding protein and ion channels, thus they are involved in the following biological processes: transcription regulation, neurogenesis and differentiation (Additional file 1: Fig. S3B, C). According to these functional characteristics most of the altered genes are mainly expressed in brain (Additional file 1: Fig. S3D). The same analysis was performed with the genes that were associated with hypomethylated events. The results showed that the most affected pathways were: MAPK, PI3K-Akt, calcium and cAMP signalling pathway, focal adhesion, neuroactive ligand-receptor interaction, inflammatory bowel disease and cancer pathways (Additional file 1: Fig. S3A). Proteins encoded by these genes are involved in cell adhesion, differentiation, neurogenesis and potassium transport. Their molecular function is mainly carried out during development and as ion channels (Additional file 1: Fig. S3B, C). Also in this case the altered genes are mostly expressed in brain (Additional file 1: Fig. S3D).

Of note, considering only genes associated with altered CpG sites within NRSE we obtained similar results but with a lower statistical significance given the reduced power of the analysis (Additional file 1: Fig. S4).

### NRSE methylation alterations in biliary tract cancer

The same methylome analysis was performed using 17 BTC and nine controls (BTC Discovery dataset) and it detected 4906 altered CpGs (2529 hypermethylated and 2377 hypomethylated) in 4792 NRSE regions using a nominal *p* value < 0.05 (Fig. 2). We found 1635 CpGs within islands, 1478 in CGI flanking regions and 1793 in open areas.

The altered CpGs validated in TCGA-CHOL were 3784 associated with 3798 NRSE regions (Fig. 2).

BTC gene enrichment analysis showed affected pathways, biological processes and molecular function similar to those found in CRC (Additional file 1: Fig. S5).

### NRSE methylation alterations in chronic lymphocytic leukemia

We explored the DNA methylation pattern of NRSE regions in 18 CLL and 6 normal samples (CLL Discovery dataset) and we detected 1393 altered CpGs (148 hypermethylated and 1245 hypomethylated) in 1750 NRSE regions (Fig. 2). We detected 197 CpGs in CGI, 608 in flanking regions and 588 in open sea.

We successfully cross-validated in an external dataset 1032 altered CpGs associated with 1323 NRSE regions (Fig. 2).

CLL gene enrichment analysis presented altered biological functions different from CRC and BTC results. Given the lower number of hypermethylated events in CLL, the functional annotation analysis did not show statistically significant results for KEGG pathway and tissue expression (Additional file 1: Fig. S6A, D). The associated genes mainly encode developmental and DNA-binding proteins, involved in transcription regulation (Additional file 1: Fig. S6B, C). The analysis performed with the genes associated with hypomethylated events showed that the only statistically significant affected pathways were: MAPK signalling and Epstein-Barr virus infection (Additional file 1: Fig. S6A). The altered genes, with a molecular function of repressor and involved in endocytosis (Additional file 1: Fig. S6B, C), are mainly expressed in blood and cervix carcinoma (Additional file 1: Fig. S6D).

### Alterations shared between PA discovery and the other analysed tumour types

**Fig. 4:**
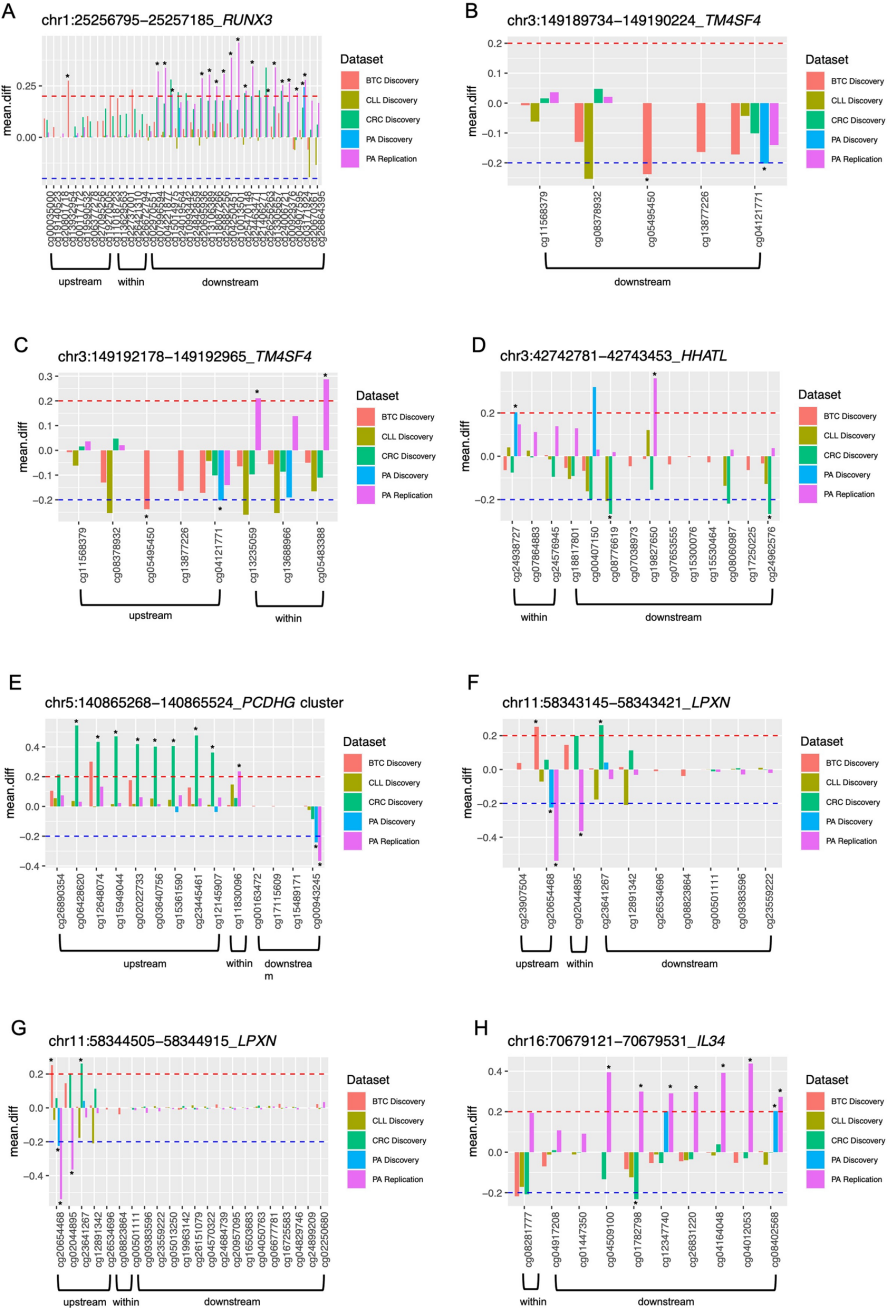
NRSE alterations shared between PA Discovery and the other analysed datasets, BTC, CLL and CRC Discovery and PA Replication. Bar plots representing the differential methylation value between tumour and normal samples of the selected NRSE regions associated with the following genes: *RUNX3* (**A**), *TM4SF4* (**B**, **C**), *HHATL* (**D**), *PCDHG* cluster (**E**), *LPXN* (**F**, **G**) and *IL34* (**H**). Asterisks indicate significantly altered and validated CpGs

**Fig. 5:**
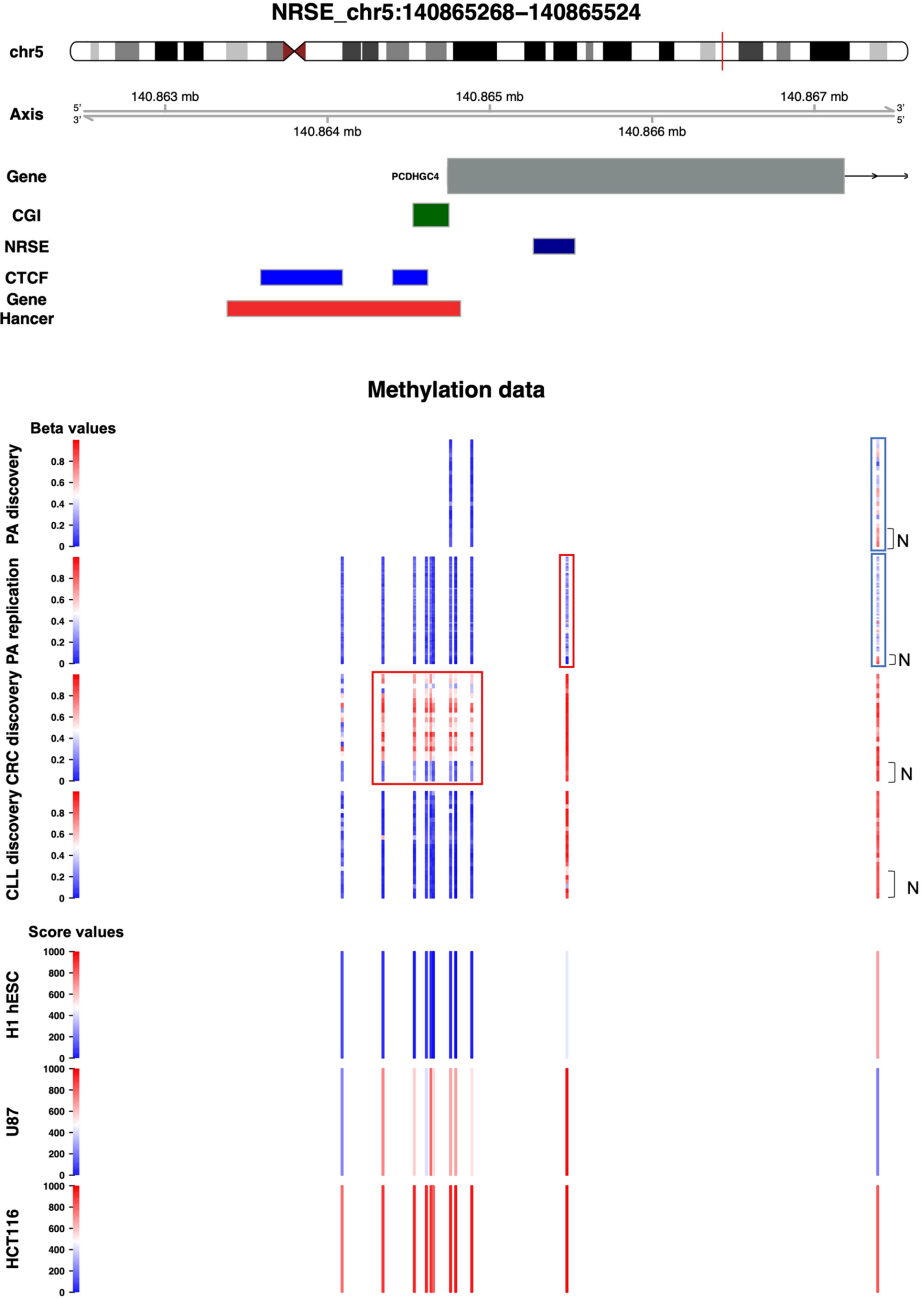
NRSE region associated with *PCDHG* cluster. Genomic localization of *PCDHG* cluster showing only an exon of one gene member, *PCDHGC4*, including the localization of CGI (annotated with the UCSC CGI names) in green box, NRSE in blue box, CTCF binding sites in light blue boxes and Gene Hancer (promoter/enhancer) in red box. Altered regions are enclosed in red (hypermethylation) and blue (hypomethylation) boxes. The figure shows the *β* values of tumour and normal samples in PA Discovery and Replication, CRC and CLL Discovery datasets, and the methylation score values of the cell lines H1 hESC, U87 and HCT116

Considering the low coverage of 27 K array used in the PA Discovery study, we first searched for shared NRSE alterations between PA discovery and the other analysed tumour datasets (CRC, BTC and CLL Discovery datasets, successfully validated). The analysis showed eight altered NRSE regions summarized in Fig. 4A–H and Additional file 1: Table S1. Given the opposite role of REST in neuronal and non-neuronal tissues, five altered NRSE regions associated with four genes, *LPXN*, *IL34*, *HHATL* and *PCDHG* cluster, resulted particularly interesting. The characteristics of these regions were: an opposite differential methylation pattern between PA Discovery and the other cancers and at least one altered CpG in PA Discovery dataset with similar methylation pattern in PA Replication dataset (Fig. 4D–H and Additional file 1: Table S1). Since REST mainly regulates neuronal genes, we focused on one NRSE region associated with *PCDHG* cluster (NRSE at chr5:140,865,268–140,865,524) (Fig. 5). The 27 K array interrogates three CpG sites in the NRSE region but only one, distant almost 2 kb from NRSE (cg00943245), resulted hypomethylated in the PA Discovery dataset. In PA Replication dataset there were 11 CpG sites interrogated in the same region. We confirmed the same alteration and we found hypermethylation at cg11830096 located within the NRSE. Of note, there was no difference in the methylation values among normal samples and among tumours from both datasets (Additional file 1: Fig. S7A, B). Instead, in CRC discovery dataset the region altered in PA is not affected but the region upstream *PCDHGC4* was hypermethylated (Additional file 1: Fig. S7C). Of note, this region overlaps with a CGI and promoter/enhancer region (Fig. 5 and Additional file 1: Fig. S7C). In CLL and BTC we did not find any alterations (Figs. 4E and 5 and Additional file 1: Table S1). We also explored the methylation pattern of this region in an embryonic cell line given the well-known role of REST in embryonic stem cells, and in two tumoral cell lines. The human embryonic stem cell line, H1 hESC, displays an unmethylated state upstream the NRSE, a partially methylated state within NRSE and methylated downstream, the U87 glioma cell line shows methylated state within and upstream NRSE while partially methylated downstream, and the colon cancer cell line, HCT116, presents an extended methylation in NRSE region (Fig. 5).

### Alterations shared between PA Replication dataset and the other analysed tumour types

**Fig. 6:**
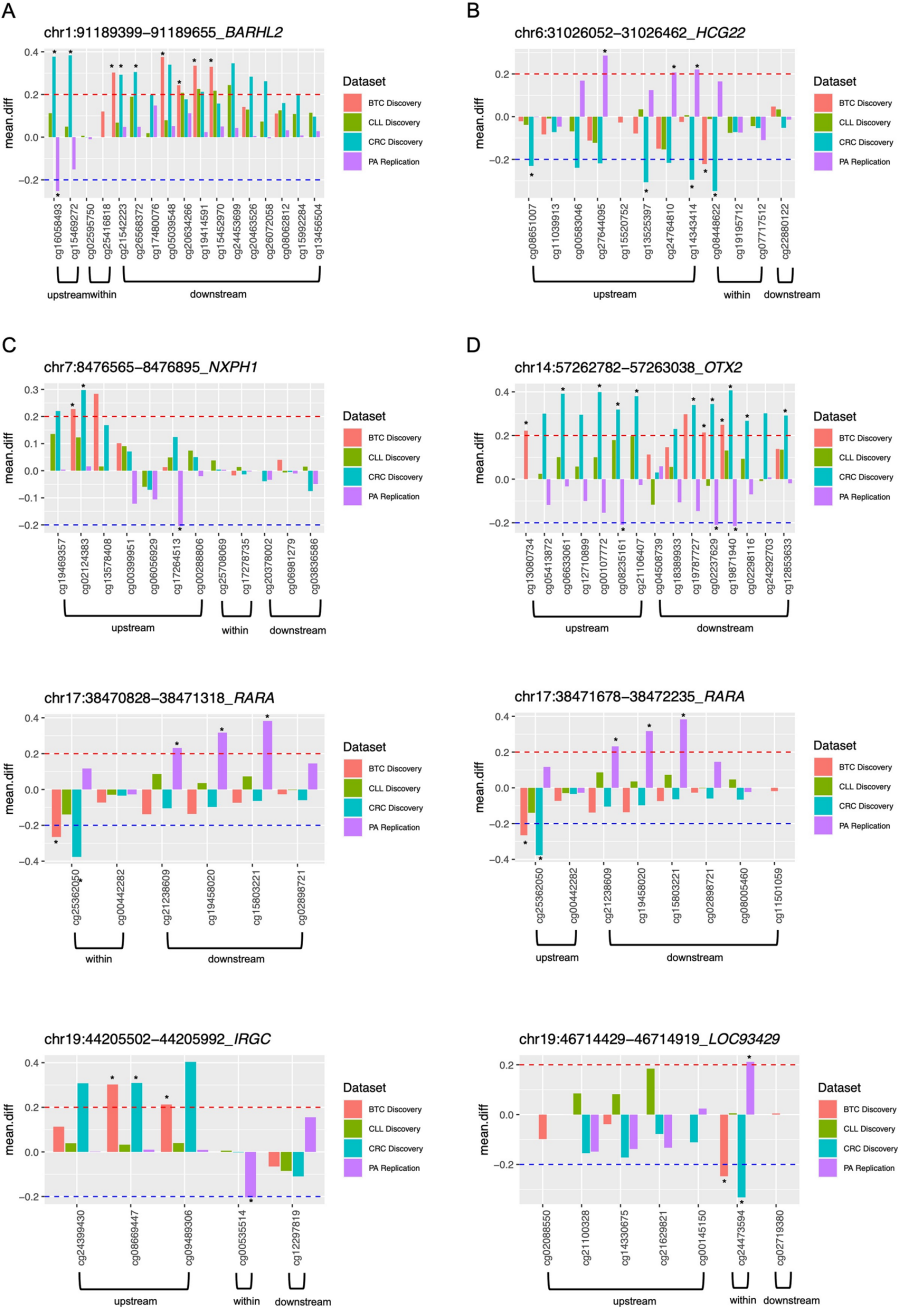
NRSE alterations shared between PA Replication and the other analysed datasets, BTC, CLL and CRC Discovery. Bar plots representing the differential methylation value between tumour and normal samples of the selected NRSE regions associated with the following genes: *BARHL2* (**A**), *HCG22* (**B**), *NXPH1* (**C**), *OTX2* (**D**), *RARA* (**E**, **F**), *IRGC* (**G**) and *LOC93429* (**H**). Asterisks indicate significantly altered and validated CpGs

**Fig. 7:**
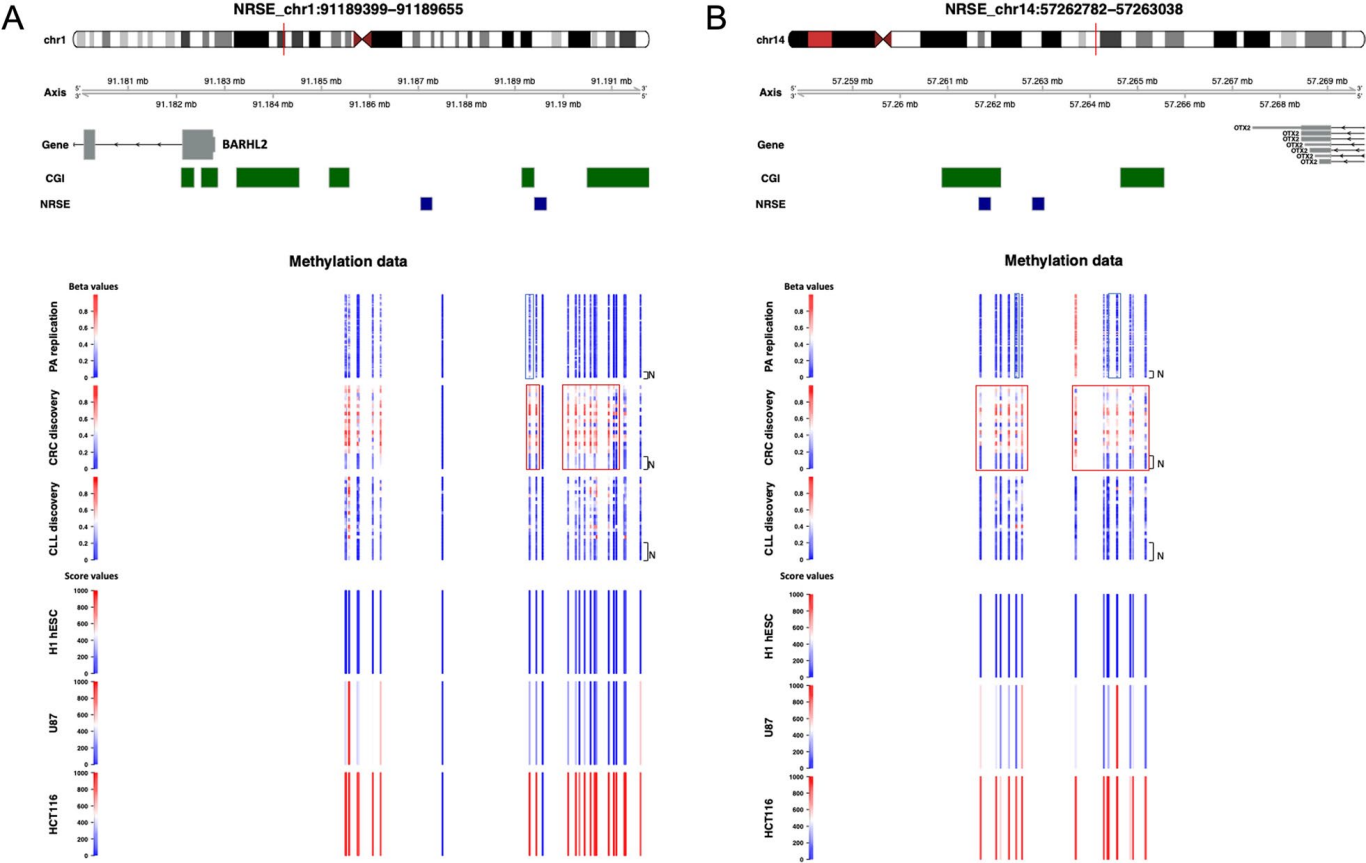
NRSE region associated with *BARHL2* (**A**) and *OTX2* (**B**). Genomic localization of *BARHL2* showing two exons (**A**) and of *OTX2* showing only one exon (**B**), including the localization of CGI (annotated with the UCSC CGI names) in green box and NRSE in blue box. Regions including altered CpGs are enclosed in red (hypermethylation) and blue (hypomethylation) boxes. The figure shows the β values of tumour and normal samples in PA Replication, CRC and CLL Discovery datasets, and the methylation score values of the cell lines H1 hESC, U87 and HCT116

Since the 27 K array used for PA Discovery methylome study has a low coverage, and thus few altered CpG sites were detected, we further explored possible opposite alterations between PA Replication set (PA Validation dataset including only the normal samples from children donors) and gastrointestinal tumours, with at least one altered CpG in common between CRC and BTC, and CLL (Fig. 2). We identified 16 altered NRSE regions (Fig. 6 and Additional file 1: Fig. S8) of which four have the same CpGs showing an opposite Δ*β* values (hypermethylated in one case and hypomethylated in the other and vice versa) in gastrointestinal tumours and PA (Fig. 6 and Additional file 1: Table S2). One of these NRSE regions, NRSE at chr19:46,714,429–46,714,919, is associated with *LOC93429*, a long non-coding RNA, whereas the NRSE located at chr6:31,026,052–31,026,462 is associated with the pseudogene HLA Complex Group 22 (*HCG22*). The other two NRSE regions, NRSE at chr1:91,189,399–91,189,655 and chr14:57,262,782–57,263,038, are associated with genes that mediate neuronal processes, BarH Like Homeobox 2 (*BARHL2*) and Orthodenticle Homeobox 2 (*OTX2*). *BARHL2*-associated NRSE is located upstream the gene, a genomic region with several CGIs and another NRSE. We detected an opposite methylation pattern between PA and CRC in the upstream area of the altered NRSE. No alteration was detected in the NRSE itself in all tumours analysed, while an extended downstream hypermethylation was found in gastrointestinal tumours (Fig. 7A). Regarding the methylation status of the selected cell lines, H1-hESC display a low methylation level across the entire NRSE region, both U87 and HCT116 show low methylation in the NRSE, while intermediate (in U87 cell lines) and high (in HCT116 cell lines) methylation levels were observed in the flanking regions (Fig. 7A). *OTX2*-associated NRSE region is located downstream the gene in a region with two CGI and another NRSE site, not selected since there were not CpGs in common between BTC and CRC. The flanking regions of *OTX2*-associated NRSE showed a constant and extensive opposite differential methylation pattern between PA and CRC, including BTC for some CpGs, being hypomethylated in PA and hypermethylated in CRC (Figs. 6 and 7B). The cell lines show a methylation pattern similar to that observed for *BARHL2*-associated NRSE region, with low methylation level for H1-hESC, intermediate level for U87 and high methylation for HCT116 (Fig. 7B). Other three NRSE regions are interesting to mention since the two associated genes are involved in neuronal mechanisms (Fig. 6 and Additional file 1: Table S2). One of these genes is neurexophilin (*NXPH1*) whose associated NRSE region, NRSE at chr7:8,476,565–8,476,895, showed an opposite DNA methylation pattern between PA and gastrointestinal tumours along the upstream flanking region. The other two NRSE regions, NRSE at chr17:38,470,828–38,471,318 and chr17:38,471,678–38,472,235, are associated with *RARA*, encoding for the Retinoic Acid Receptor Alpha. They present hypomethylated CpG sites within and upstream the NRSE in CRC and BTC, whereas in PA we detected hypermethylation downstream. Another NRSE region, NRSE at chr19:44,205,502–44,205,992, interesting to mention is associated with an enhancer and a CGI but the nearest gene, Interferon-Inducible GTPase 5 (*IRGC*), is located at 16464 bp downstream. In PA we detected hypomethylation within NRSE but no alteration was observed in gastrointestinal tumours that showed alteration in the upstream region (Fig. 6 and Additional file 1: Table S2).

### Chromatin status within selected NRSE regions

We explored the presence of transcription factors binding sites within the altered NRSEs associated with *BARHL2*, *OTX2* and *PCDHGC4* in publicly available data of H1 hESC since REST signal was detected in this cell line. As observed in the Additional file 1: Table S3, we found several binding sites of transcription factors involved in REST regulation mechanism, such as SIN3A and HDAC recruited by REST. We also checked the methylation and acetylation status of histones and observed a pattern linked to transcriptional inactivation as supported by the Chromatin State Segmentation data, in agreement with REST-mediated neuronal gene silencing in stem cells (Additional file 1: Table S4).

## Discussion

REST is a silencing transcription factor that mediates the regulation of neuronal genes during development and in terminally differentiated non-neuronal cells. In differentiated non neuronal cells, REST binds to its recognition motifs and induce long-term silencing of target genes, whereas in embryonic stem cells and neuronal progenitors, the target genes are in a permissive state leading to low but detectable transcript levels. Instead, in mature neurons, REST is downregulated, thus its binding sites are not occupied and chromatin is in a relaxed state allowing gene transcription [11]. Since REST orchestrates chromatin modification of target regions including DNA methylation and it has been discovered that REST is dysregulated in cancer [24–27, 29], we wonder whether DNA methylation pattern of NRSE and its flanking regions is altered in tumours probably contributing to the alteration of REST-mediated gene regulation. Our methylome analyses showed several altered NRSE regions in both neuronal and non-neuronal genes in the analysed tumours, including a type of brain tumour, PA, two types of gastrointestinal cancers, CRC and BTC, and a blood cancer, CLL. This agrees with the well-known role of REST in neuronal gene regulation, however recent studies reported that a low percentage of neuronal genes contain NRSE [36]. In fact, Otto and colleagues found that REST binding motifs are located not only in neuronal genes but also in individual members of large gene families, such as voltage-dependent ion channels, in genes associated with cell adhesion, such as protocadherins, and in genes involved in immune response [37]. This evidence is in accordance with the results of our functional analyses. CLL was mostly characterized by alteration in immune-related genes. On the other hand, in gastrointestinal tumours it was observed an enrichment of aberrantly methylated NRSE regions in pathways related to transcription regulation, neuronal activities and cell differentiation. Our previous work has already reported that DNA methylation alterations affect genes associated with neuron-related pathways and cell crosstalk suggesting a functional involvement in colon cancer [1] that was also supported by other authors [35].

To improve the validity of our findings, we replicated our results in publicly available datasets. However, as described in the results, we could not successfully validate many alterations obtained in the PA Discovery. These conflicting results may be due to the differences between normal samples. On one hand, commercial normal samples from the validation dataset belong to subjects with different range of ages; children below 10 months of birth and adults with 33 and 87 years old. It is known that DNA methylation pattern changes during the lifespan of the cell. DNA methylation level can be used as a biomarker to predict human ageing, i.e. a molecular clock. To this aim, ageing models based on DNA methylation status at specific CpG sites were built to address developmental biology, disease onset and ageing [38–41]. Specifically, DNA methylation pattern changes during brain development in perinatal stage following a methylation/demethylation program. The accumulation of DNA methylation alterations during human lifespan contributes to an increased risk of age-related diseases including neurodegenerative pathologies [38, 41]. In fact, alterations in methylation may also contribute to the diagnosis, prognosis and clinical outcome of neurological disorders [42, 43]. Considering the relevance of REST during neurogenesis, cell differentiation and its expression changes with age [9, 22], different methylation pattern in NRSE regions among groups of age could be expected. In fact, methylation β values of adult samples differ from those corresponding to children samples, suggesting that NRSE regions methylation pattern may also change with age. On the other hand, previous research reported REST subcellular mislocalization and overexpression in epilepsy, leading to its abnormal function and the consequent channels and signalling proteins expression dysregulation [44–47]. Given this evidence, it can be expected that REST binding sites are aberrantly methylated in epileptic subjects reinforcing its dysfunction. In our PA Discovery dataset, control samples obtained from epileptic subjects present a different methylation pattern respect to the normal samples of the PA Validation dataset. To the best of our knowledge, this is the first work that reports NRSE methylation alterations according to age and epilepsy. Interestingly, a differently methylated NRSE between epileptic and non-epileptic individuals is associated with *DHX58* gene, belonging to the *DHX* gene family that is involved in epilepsy. In particular, a de novo pathogenic *DHX58* variant has been recently reported [48, 49]. The identified alteration overlaps with the first exon of *DHX58* and may alter its transcription contributing to its aberrant function. Another NRSE region altered in epileptic samples is associated with *HAAO*. This gene encodes an enzyme involved in the synthesis of quinolic acid (QUIN), a neurotoxin that plays a role in the pathogenesis of neurologic disorders given its association with seizure mechanism [50–52]. The identified hypomethylation event at NRSE region may alter REST-mediated regulation of *HAAO* impacting on QUIN production. To note, the methylation level in these NRSE regions, associated with *DHX58* and *HAAO*, was homogeneous among children and adult control samples from PA Validation, reinforcing the possible link of the identified alterations with epilepsy. These results may attract the interest to perform further DNA methylation studies at NRSE in other neuronal pathologies.

Considering the age of tumour and normal samples, we continued the analysis using only the children control samples from the PA Validation dataset as an independent cohort, defined as PA Replication. To note, the analysis of this dataset revealed much more aberrations in PA (14,416 altered NRSE) respect to gastrointestinal tumours (approximately 4600 altered NRSE) and CLL (1750 altered NRSE), probably due to REST overexpression in brain tumours, contributing to the dysregulation of REST-mediated mechanism [22, 23].

Given the different role of REST in neuronal and non-neuronal tissues, we focused on alterations with opposite DNA methylation pattern between PA Discovery and the other analysed tumours. We did not find alterations shared between PA and CLL, while eight NRSE regions were altered in both PA and gastrointestinal tumours. One of the altered NRSE region was associated with *PCDHG* cluster, a gene cluster with a known essential role in nervous system that has been found frequently altered at DNA methylation and expression level in solid tumours [30, 53–55]. We detected higher methylation level upstream NRSE in CRC and HCT116 colon cell line than in control tissue. On the other hand, PA displays higher methylation values within NRSE and lower downstream than controls, in accordance with the levels observed in U87, a glioma cell line. We also considered the methylation of an embryonic cell line, H1 hESC, showing a methylation level that increases from downstream to upstream the NRSE. To note, Encode data showed that REST binds to this NRSE in H1 hESC. These results support a tumour tissue-specific trend of DNA methylation changes at NRSE region and suggest a different REST-mediated regulation agreeing with its distinct role in stem cells, nervous and non-nervous system.

Among the other altered NRSE regions not associated with neuronal genes, it is interesting to mention that the altered NRSE associated with *HHATL* gene overlaps with an enhancer/promoter region. *HHATL* encodes a hedgehog acyltransferase, mainly expressed in brain, heart, skeletal muscle and thyroid. Ehrlich and collaborators highlight the relevance of chromatin structure for the transcription regulation of *HHATL* and its neighbouring gene *KLHL40*, a *KLHL* family member. Both genes are located in the same topologically associating domain, where long-distance interactions between their enhancer/promoter have been demonstrated [56]. Given this common mechanism of *HHATL* and *KLHL40* modulation through enhancers, highly important in establishing tissue-specific expression, the alterations detected may potentially contribute to their dysregulation. This evidence further supports the involvement of REST in the regulation of an expanded area, although additional experiments are needed to elucidate whether DNA methylation alterations at NRSE overlapping with enhancer may affect REST binding, loop formation and transcription. To support REST involvement in *HHATL* and *KLHL* regulation, previous studies suggest that smooth muscle- and heart-specific genes may be regulated by REST [57–61].

We continued our comparison analysis between brain and non-brain tumours using the PA Replication dataset since it has a higher coverage than PA Discovery dataset, finding 16 NRSE regions. Interestingly, two of these NRSE regions are associated with homeobox genes, *BARHL2* and *OTX2*, that have a crucial role in neurological processes. Similarly to NRSE-associated with *PCDHG* cluster, it was demonstrated that these two NRSEs are bound by REST in H1 hESC. *BARHL2* encodes for BarH Like Homeobox 2, mainly expressed in brain and involved in neuron generation and axon extension. It is important to mention that *BARHL2* has been found aberrantly methylated in epithelial and hematological tumours as well as brain tumours [62–66] and its putative role as tumour suppressor has been suggested in gastric cancer [65]. In our analysis, the NRSE is not methylated in both CRC and PA but the methylation differences are observed in the flanking regions. CRC and HCT116 cell line display higher methylation level in NRSE flanking regions than normal samples, while PA is hypomethylated upstream with similar methylation level to U87 cell line. Instead, an embryonic stem cell line presents an unmethylated state. *OTX2*, Orthodenticle Homeobox 2, encodes for a protein that acts as a transcription factor, mainly involved in brain, craniofacial and sensory organ development, also influencing the proliferation and differentiation of dopaminergic neuronal progenitor cells. Similarly to *BARHL2*, *OTX2* has been found aberrantly methylated in epithelial tumours including breast and lung cancer [66, 67]. An oncogenic role for OTX2 has been demonstrated in the proliferation and progression of medulloblastoma in which OTX2 induces epigenetic modifications restoring a stem cell-like phenotype [68]. Our results show an expanded methylation alteration at NRSE flanking regions, hypermethylated in gastrointestinal tumours and hypomethylated in PA. The cell lines show a similar methylation trend to tumour tissue samples, whereas H1 hESC is unmethylated at the identified NRSE region. Interestingly, the hypomethylation observed in PA may be a mechanism to restore the methylation pattern typical of embryonic stem cell, potentially leading to OTX2 upregulation and triggering a stem cell-like phenotype. It is important to consider that in non-neuronal tumours, although loss of function of REST has been frequently reported in cancer, this not always lead to the de-repression of its target genes, suggesting an even more complex mechanism [37].

To note, it is important to consider that REST regulation mechanism involved several factors to model chromatin architecture of the target region. Our results, focused on the exploration of selected NRSE regions in H1 hESC, show that REST binding at NRSE overlaps with the presence of other transcription factors and chromatin remodelers, acting in concert to determine a specific epigenetic pattern.

Among the altered NRSE regions that are not associated with neuronal genes, we mention the one overlapping with an enhancer within the gene body of *HCG22*. It is a pseudogene that belongs to a mucin-like gene cluster including *MUC22*, *MUC21*, and *DPCR1* on chromosome 6p21.3 [69]. It has also a role as a non-coding RNA, altered in several cancer types [70–72]. Given the complex regulation mechanism of gene cluster, the presence of altered NRSE regions, as observed for *PCDHG* and mucin-like clusters, may dysregulate gene expression in these expanded areas.

## Conclusions

The DNA methylation aberrations found at NRSE regions may agree with a distinct REST dysregulation mechanism in brain and non-brain tumours given the opposite REST expression level and the presence of *REST* splice variants. The impact of these methylation alterations at NRSE site and flanking regions on REST binding needs to be further addressed. Our results and previous evidence suggest REST as a probable master regulator of specific-gene expression, acting through epigenetic remodeling. Therefore, cancer methylation aberrations, frequently early events, may be the consequence of its dysfunction. It is intriguing to consider the possibility of therapeutic interventions for the epigenetic reprogramming of REST targets based on the modulation of this master regulator to restore its functionality and the normal cell state.

## Methods

### Experimental discovery datasets

Our experimental discovery datasets included DNA methylation data of one brain cancer (PA), two gastrointestinal tumours (CRC and BTC) and one blood cancer (CLL), previously published [1, 30, 31, 34, 73] (Additional file 1: Fig. S1). We analysed the following:

- 20 paediatric PAs and four normal brain control samples obtained from adult epileptic individuals;
- 18 primary CRC and four matched normal samples;
- 17 BTCs whose nine paired tumour and normal samples;
- 18 CLLs and six normal blood control samples.

### Validation datasets

The following datasets were used to validate the methylation alterations detected in our Discovery datasets (Additional file 1: Fig. S1). GSE44684 (61 PA and 6 normal controls) Illumina 450 K methylation data were downloaded from the NCBI Gene Expression Omnibus Portal [74]. We also use this dataset including only children controls as an independent cohort defined as a PA Replication dataset. Illumina 450 K methylation data from The Cancer Genome Atlas (TCGA), including colon adenocarcinoma (TCGA-COAD, including 313 tumour and 38 normal controls) and cholangiocarcinoma (TCGA-CHOL, comprising 36 tumour and nine normal controls) were downloaded using the Bioconductor package TCGAbiolinks [75]. ICGC: CLLE-ES (139 tumours and 20 normal controls) Illumina 450 K methylation data were obtained from the International Cancer Genome Consortium (IGCG) Data Portal [76].

Data were analysed following the same pipeline used for the Discovery data.

Methylation data of H1-hESC (human embryonic stem cells), U87 (human glioblastoma) and HCT116 (human colon cancer) cell lines were obtained from UCSC HAIB Methyl450 Track.

### NRSE and methylation analysis

NRSE genomic coordinates were obtained from ENCODE 3, including Transcription Factor ChIP-seq Peaks of 338 factors in 130 cell types, downloaded from the University of California Santa Cruz (UCSC) Genome Browser. We also integrated U87 cell line information from the previous ENCODE (Transcription Factor ChIP-seq Uniform Peaks), downloaded from UCSC. Since REST may also affect DNA methylation at sequences adjacent to NRSE, we investigated the methylation status of CpGs in an extended area defined as NRSE region considering a distance of 2000 bp from NRSE.

Illumina methylation data, obtained from 27 K array (PA), 450 K array (CRC and CLL) and 850 K array (BTC), were analysed as previously described [1, 30, 31, 34, 73]. Briefly, a differential methylation analysis at CpG level was performed between tumour and normal samples from each dataset using the limma method [77]. P values are computed for each CpG site and corrected for multiple testing using the false discovery rate (FDR) method. CpGs were annotated according to the respective Illumina Manifest and associated with the nearest genes and transcripts using R annotation package FDb.InfiniumMethylation.hg19 [78]. We included in the analysis whether the CpG site was located in enhancers, CGI, CGI flanking regions or open sea areas. We selected only differentially methylated probes (Δ*β* values ≥ 0.2 or ≤—0.2, i.e. 20% differential methylation level) located in NRSE region. Hypermethylation was defined as Δ*β* values > 0.2 and adjusted *p* value < 0.05, while hypomethylation was defined as Δ*β* values < − 0.2 and adjusted *p* value < 0.05. Since the results of this analysis were less robust in BTC, we used the nominal threshold (*p* values < 0.05). Finally, the methylation value of each altered CpG for each sample of PA datasets has been used in an analysis of UHC and visualized by Bioconductor package ComplexHeatmap [79].

Enhancer data for the region of interest were obtained from GeneHancer Track, downloaded from UCSC.

Transcription Factor ChIP-seq Peaks data from ENCODE 3 were used to investigate REST interactors and other factors binding in the selected NRSE regions considering H1-hESC since REST signal was detected in this cell line.

Histone data of H1-hESC for the NRSE regions of interest were obtained from Broad Histone Track, downloaded from UCSC to investigate the epigenetic chromatin pattern.

Chromatin State Segmentation data related to the NRSE regions of interest were obtained from BroadChromHMM Track, download from UCSC.

### Gene enrichment

Functional analyses were performed using the functional annotation tool DAVID selecting Biological process, Molecular function, KEGG pathway and tissue expression [80, 81]. The study was conducted using the list of genes associated with the altered NRSE regions.

## Supplementary Information

**Additional file 1**. Suppementary material.

## Data Availability

The datasets used and/or analysed during the current study are available from the corresponding author on reasonable request.
