# Supplementary material for: DNA methylation alterations at RE1-silencing transcription factor binding sites and their flanking regions in cancer
Source: Clin Epigenetics. 2023 Jun 10;15:98. doi: 10.1186/s13148-023-01514-9 (PMC10257853; doi:10.1186/s13148-023-01514-9)
Supplement: Supplementary file 1 — Additional file 1. Suppementary material. [file 13148_2023_1514_MOESM1_ESM.doc]

DNA methylation alterations at RE1-silencing transcription factor binding sites and their flanking regions in cancer

Ana Florencia Vega-Benedetti^1,#^, Eleonora Loi^1,#^, Loredana Moi^1^ and Patrizia Zavattari^1,*^

^1^ Department of Biomedical Sciences, Unit of Biology and Genetics, University of Cagliari, 09042 Cagliari, Italy

^#^ These two authors contributed equally to the work.

^*^ Correspondence:

Prof. Patrizia Zavattari

Department of Biomedical Sciences, Unit of Biology and Genetics

University of Cagliari

Cittadella Universitaria di Monserrato, SP 8, Km 0.700

Monserrato, Cagliari, 09042, Italy

Tel: +39 070 675 4101

pzavattari@unica.it

**Supplementary Fig. 1**

**
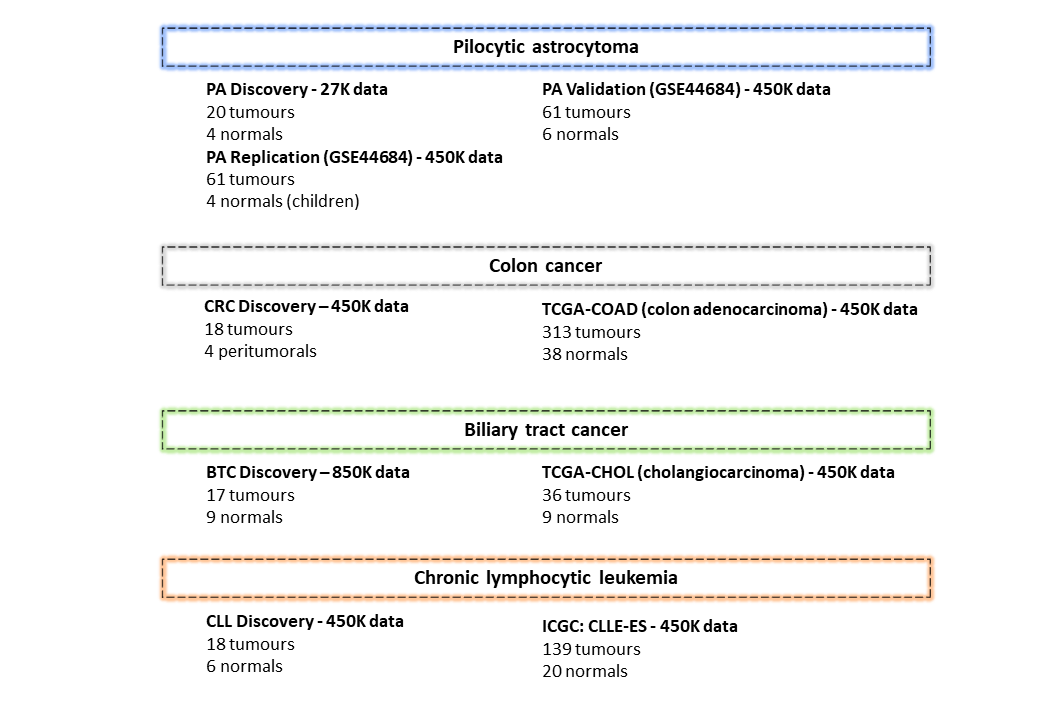
Supplementary Fig. 1**. Description of sample datasets used for the explorative and validation analyses.

**Supplementary Fig. 2**

**Supplementary Fig. 2**. Heatmap of the 120 altered CpG sites in PA Validation dataset. Unsupervised hierarchical clustering analysis based on the β value of each tumour sample.

**Supplementary Fig. 3**

**
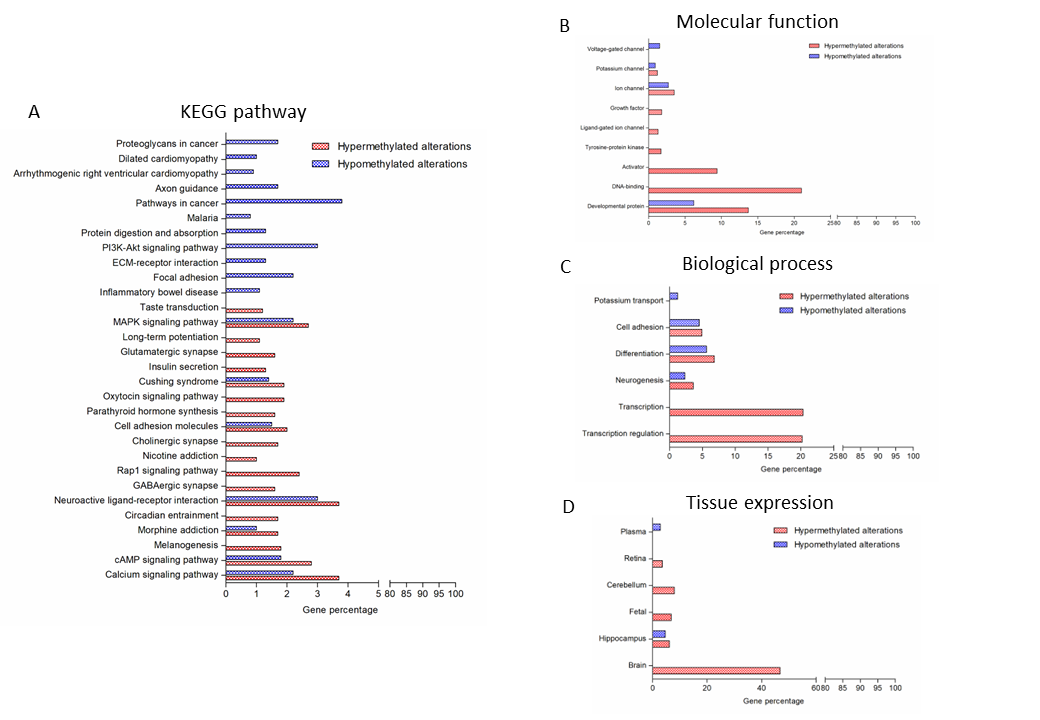
**

**Supplementary Fig. 3**. Functional annotation of methylation alterations, hypermethylated (red) and hypomethylated (blue), in CRC Discovery dataset. Bar plots showing the percentage of genes associated with KEGG pathways (A), molecular function (B), biological process (C) and tissue expression (D) with a statistically significant gene enrichment.

**Supplementary Fig. 4**


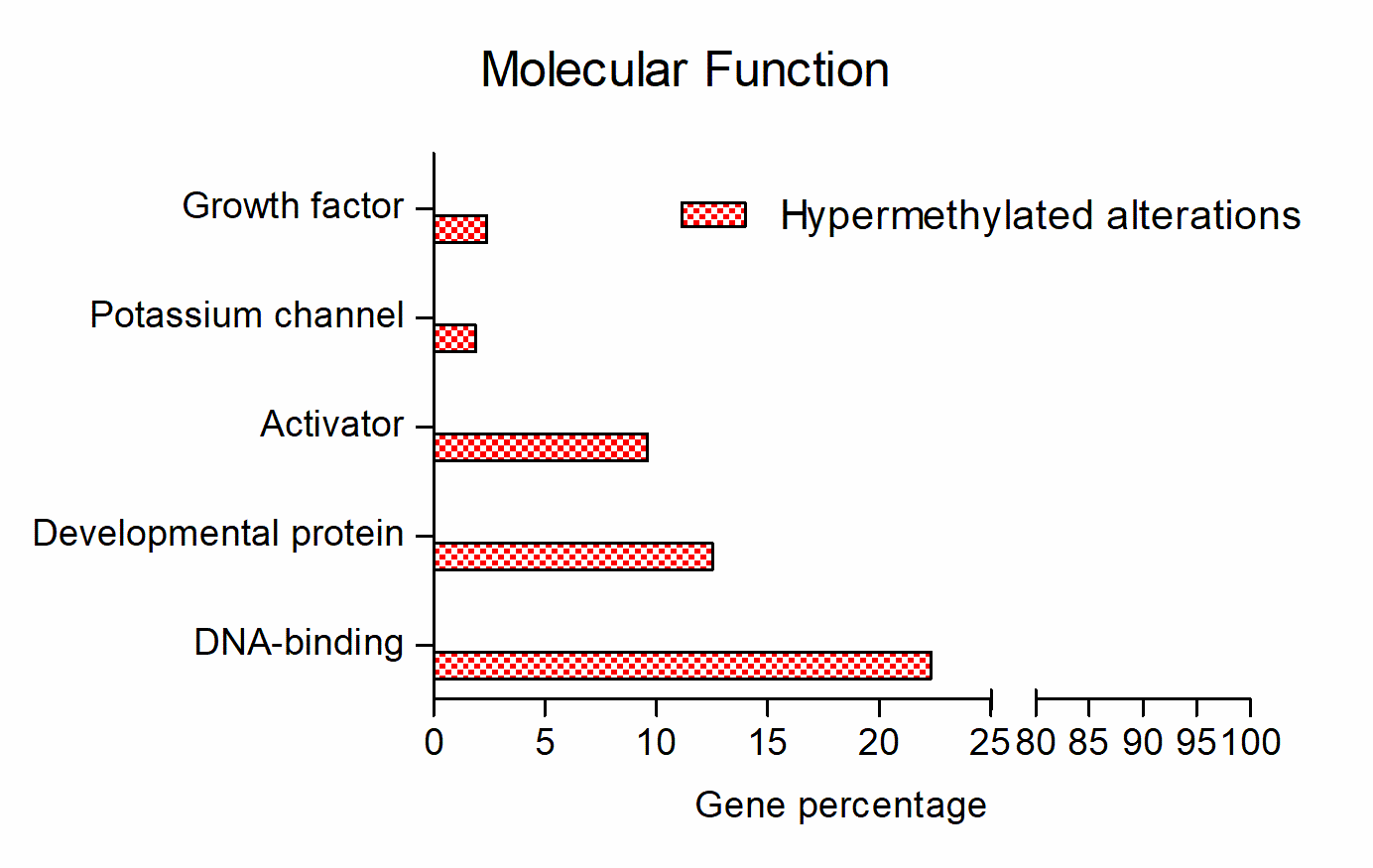

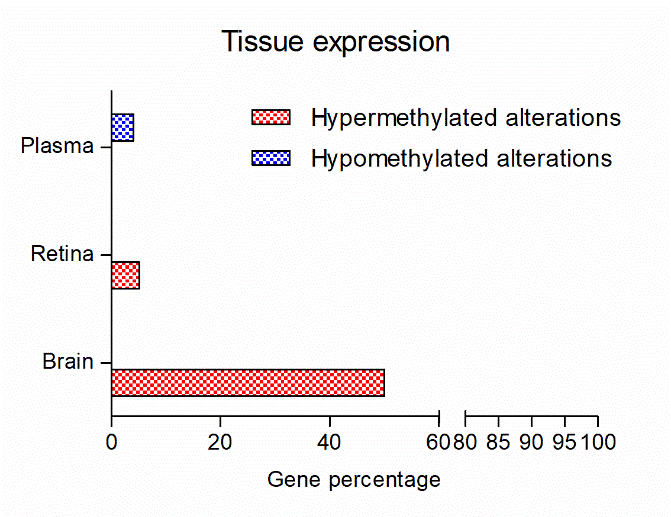

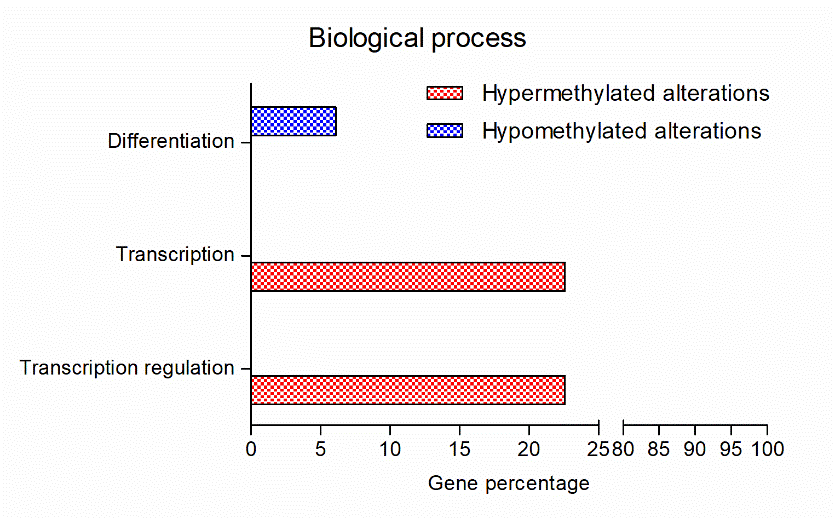

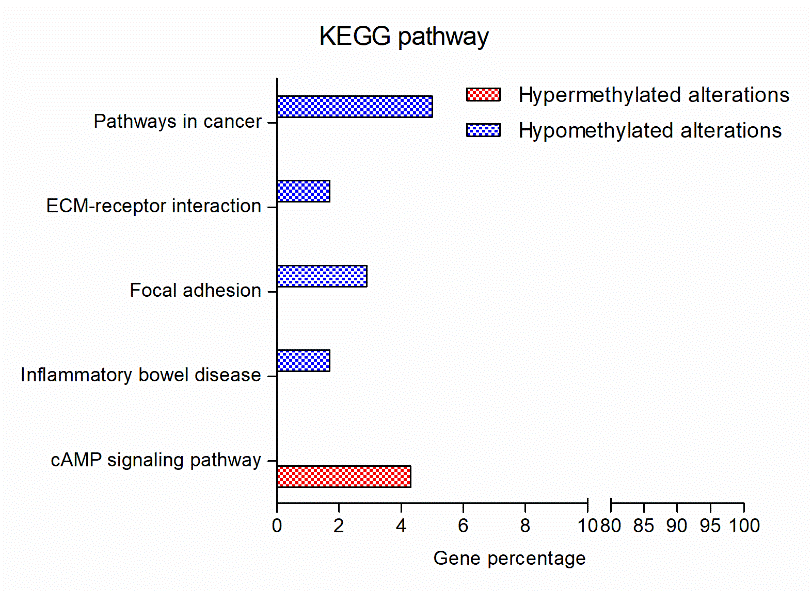


KEGG pathway

A

Molecular function

B

Biological process

C

Tissue expression

D

**Supplementary Fig. 4**. Functional annotation of methylation alterations within NRSE, hypermethylated (red) and hypomethylated (blue), in CRC Discovery dataset. Bar plots showing the percentage of genes associated with KEGG pathways (A), molecular function (B), biological process (C) and tissue expression (D) with a statistically significant gene enrichment.

**Supplementary Fig. 5**


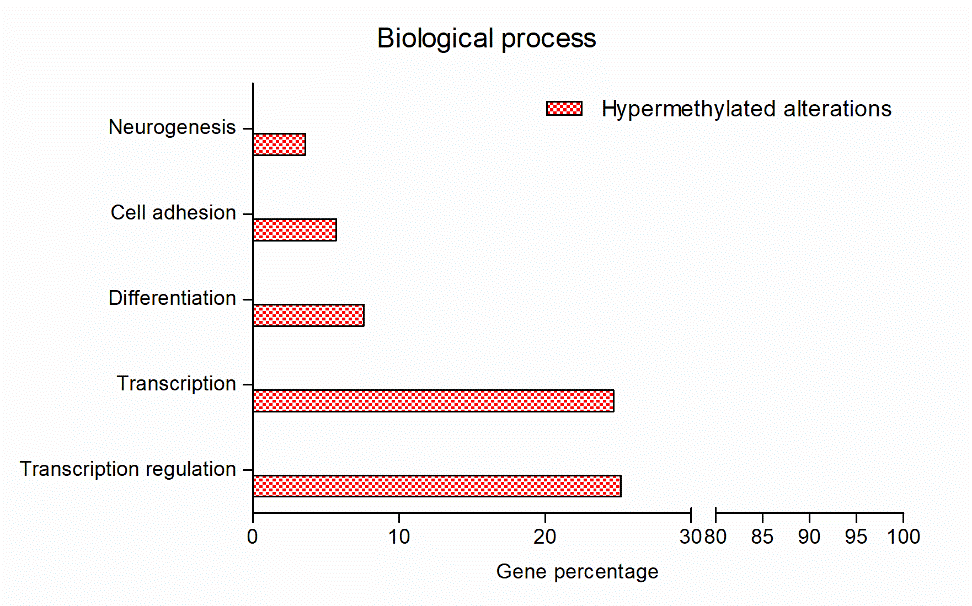

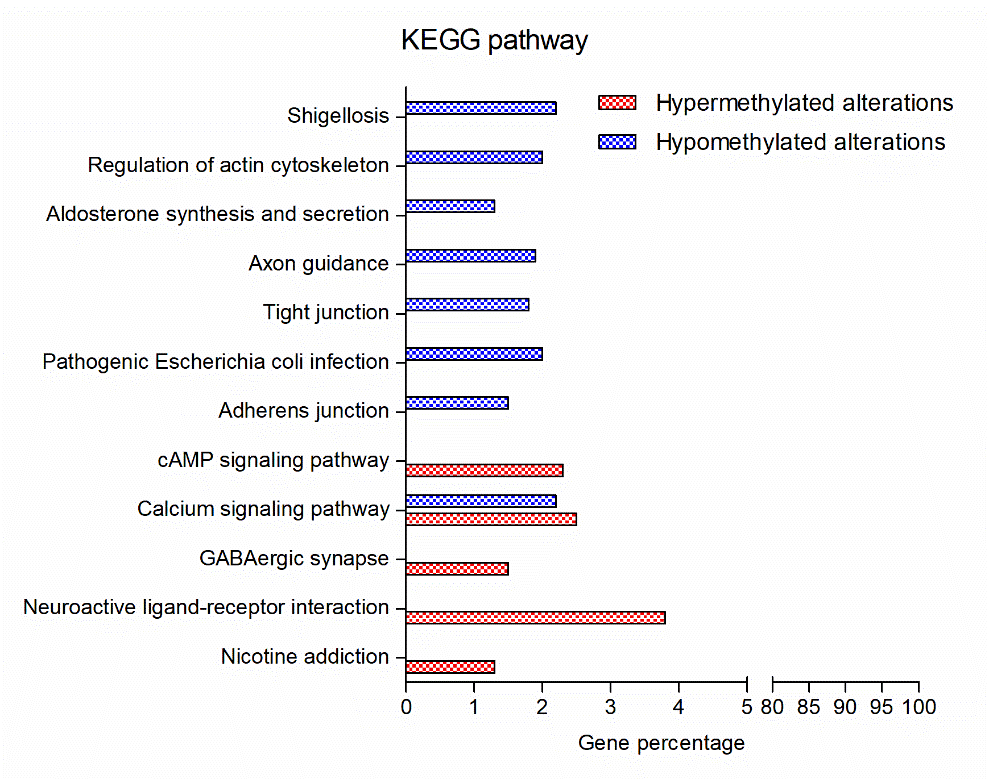

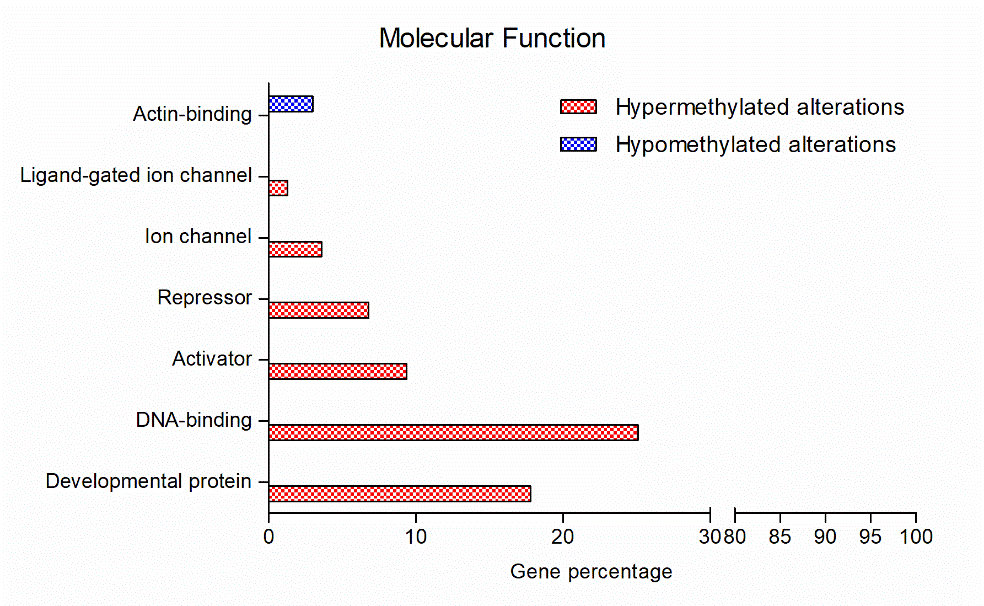

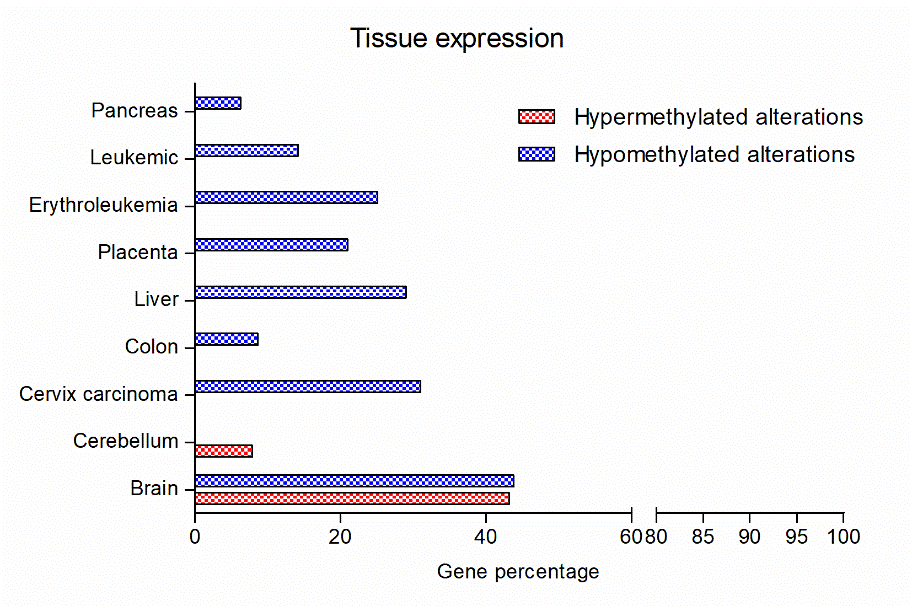


KEGG pathway

A

Molecular function

B

Biological process

C

Tissue expression

D

**Supplementary Fig. 5**. Functional annotation of methylation alterations, hypermethylated (red) and hypomethylated (blue), in BTC Discovery dataset. Bar plots showing the percentage of genes associated with KEGG pathways (A), molecular function (B), biological process (C) and tissue expression (D) with a statistically significant gene enrichment.

**Supplementary Fig. 6**

**
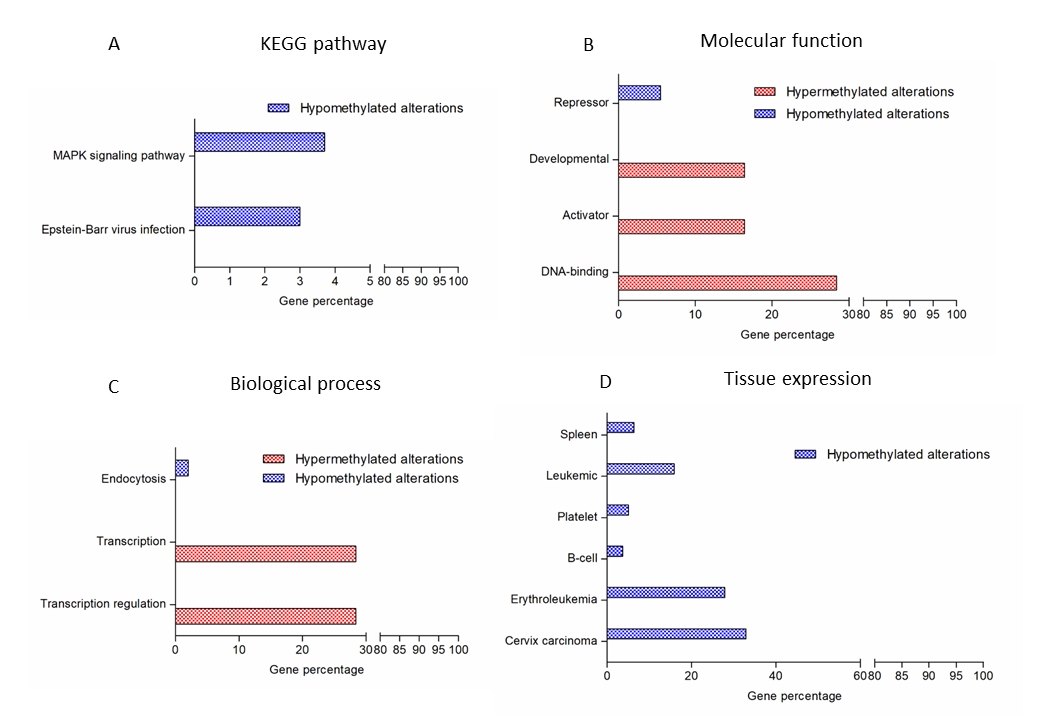
**

**Supplementary Fig. 6**. Functional annotation of methylation alterations, hypermethylated (red) and hypomethylated (blue), in CLL Discovery dataset. Bar plots showing the percentage of genes associated with KEGG pathways (A), molecular function (B), biological process (C) and tissue expression (D) with a statistically significant gene enrichment.

**Supplementary Fig. 7**


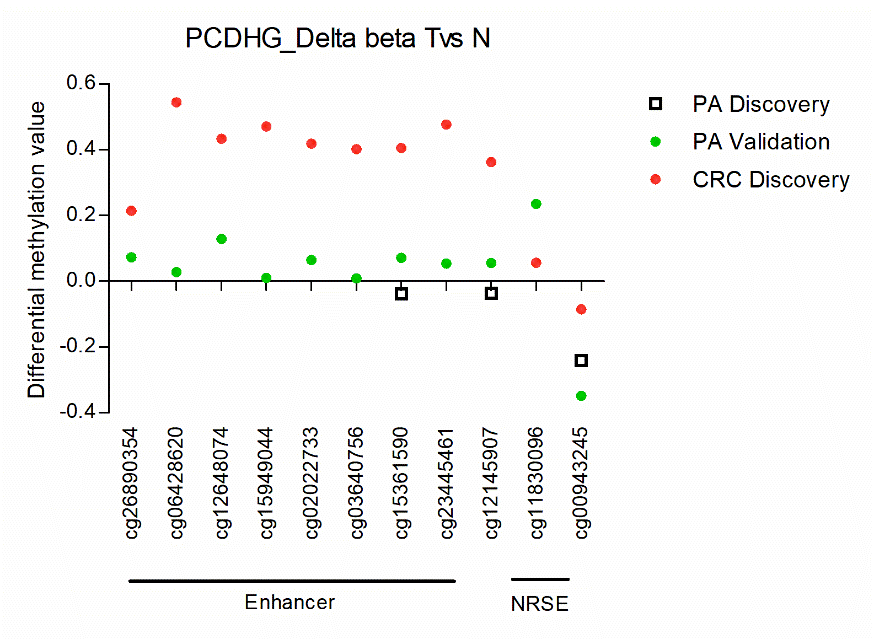

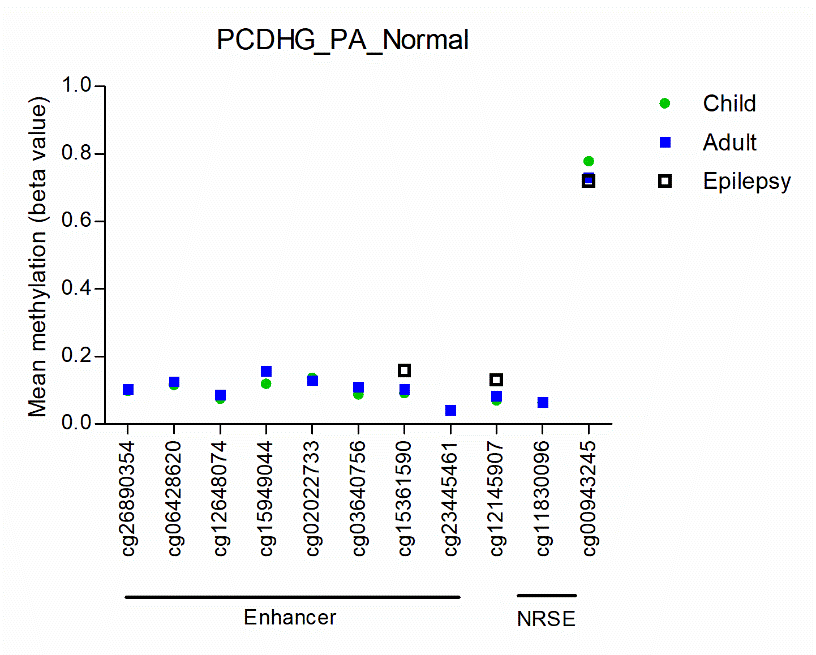

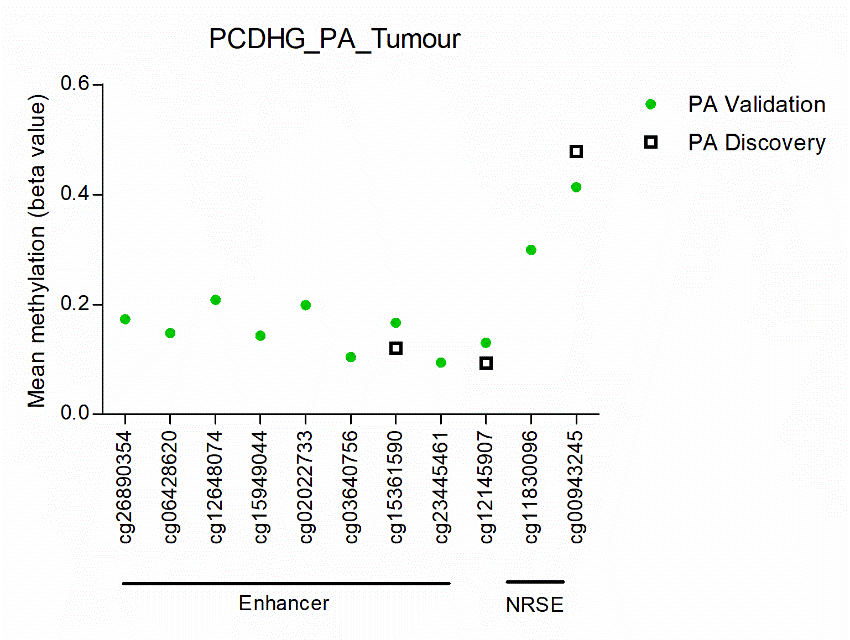


NRSE coordinates: chr5:140865268-140865524

*PCDHG cluster*

Normal Samples – PA datasets

Tumour Samples

Delta beta

A

B

C

**Supplementary Fig. 7**. DNA methylation at NRSE region associated with *PCDHG* cluster. Average β values of control (A) and tumour samples (B) from PA Discovery and Validation datasets. Differential β values between tumour and control samples from PA Discovery, PA Validation and CRC Discovery datasets (C).

**Supplementary Table 1**. Altered NRSE regions, including all interrogated CpGs, shared between PA Discovery and the other analysed datasets, BTC, CLL and CRC Discovery and PA Replication.

| **NRSE** | **CpGs interrogated** | **Relation to NRSE** | **PA Discovery** | **PA Replication** | **CRC Discovery** | **BTC Discovery** | **CLL Discovery** |
| --- | --- | --- | --- | --- | --- | --- | --- |
| **chr1:25256795-25257185 Nearest TSS gene: *RUNX3*** | cg00035000 | upstream | NA |  |  |  |  |
|  | cg19140523 | upstream | NA | NA | NA |  | NA |
|  | cg20801718 | upstream | NA |  |  |  |  |
|  | cg13932954 | upstream | NA | NA | NA |  | NA |
|  | cg00117172 | upstream |  |  |  | NA |  |
|  | cg19590532 | upstream | NA |  |  | NA |  |
|  | cg06377278 | upstream |  |  |  |  |  |
|  | cg27095256 | upstream | NA | NA | NA |  | NA |
|  | cg19270505 | upstream | NA |  |  |  |  |
|  | cg11018723 | within | NA |  |  |  |  |
|  | cg13629563 | within | NA |  |  |  |  |
|  | cg22737001 | within |  |  |  | NA |  |
|  | cg26421310 | within | NA |  |  |  |  |
|  | cg26672794 | within | NA |  |  |  |  |
|  | cg02970551 | downstream | NA |  |  |  |  |
|  | cg07996594 | downstream | NA |  |  |  |  |
|  | cg04221877 | downstream | NA |  |  |  |  |
|  | cg15014975 | downstream | NA |  |  | NA |  |
|  | cg24019564 | downstream |  |  |  |  |  |
|  | cg10993442 | downstream | NA |  |  |  |  |
|  | cg24842859 | downstream | NA |  |  |  |  |
|  | cg20695936 | downstream | NA |  |  |  |  |
|  | cg13106389 | downstream | NA |  |  |  |  |
|  | cg18087266 | downstream | NA |  |  |  |  |
|  | cg25882256 | downstream | NA |  |  |  |  |
|  | cg04250451 | downstream | NA |  |  |  |  |
|  | cg10013501 | downstream | NA |  |  |  |  |
|  | cg25470148 | downstream | NA |  |  |  |  |
|  | cg24463471 | downstream | NA |  |  | NA |  |
|  | cg21406271 | downstream | NA |  |  |  |  |
|  | cg26256263 | downstream | NA |  |  | NA |  |
|  | cg13305657 | downstream | NA |  |  |  |  |
|  | cg24006721 | downstream | NA |  |  |  |  |
|  | cg00929376 | downstream | NA |  |  | NA |  |
|  | cg04907595 | downstream | NA |  |  |  |  |
|  | cg03171924 | downstream |  |  |  |  |  |
|  | cg20670361 | downstream | NA |  |  |  |  |
|  | cg26864395 | downstream | NA |  |  | NA |  |
|  | N° of altered CpGs / interrogated CpGs | | 1/5 | 17/35 | 0/35 | 1/30 | 0/35 |
| **chr3:149189734-149190224 Nearest TSS gene: *TMS4F4*** | cg11568379 | downstream | NA |  |  |  |  |
|  | cg08378932 | downstream | NA |  |  |  |  |
|  | cg05495450 | downstream | NA | NA | NA |  | NA |
|  | cg13877226 | downstream | NA | NA | NA |  | NA |
|  | cg04121771 | downstream |  |  |  |  |  |
|  | N° of altered CpGs / interrogated CpGs | | 1/1 | 0/3 | 0/3 | 1/5 | 0/3 |
| **chr3:149192178-149192965 Nearest TSS gene: *TMS4F4*** | cg11568379 | upstream | NA |  |  |  |  |
|  | cg08378932 | upstream | NA |  |  |  |  |
|  | cg05495450 | upstream | NA | NA | NA |  | NA |
|  | cg13877226 | upstream | NA | NA | NA |  | NA |
|  | cg04121771 | upstream |  |  |  |  |  |
|  | cg13235059 | within | NA |  |  |  |  |
|  | cg13688966 | within |  |  |  |  |  |
|  | cg05483388 | within | NA |  |  |  |  |
|  | N° of altered CpGs / interrogated CpGs | | 1/2 | 2/6 | 0/6 | 1/8 | 0/6 |
| **chr3:42742781-42743453 Nearest TSS gene: HHATL** | cg24938727 | within |  |  |  |  |  |
|  | cg07864883 | within | NA |  |  | NA |  |
|  | cg24576945 | within | NA |  |  |  |  |
|  | cg18817801 | downstream | NA |  |  |  |  |
|  | cg00407150 | downstream |  |  |  |  |  |
|  | cg08776619 | downstream | NA |  |  | NA |  |
|  | cg07038973 | downstream | NA | NA | NA |  | NA |
|  | cg19827650 | downstream | NA |  |  |  |  |
|  | cg07653555 | downstream | NA | NA | NA |  | NA |
|  | cg15300076 | downstream | NA | NA | NA |  | NA |
|  | cg15530464 | downstream | NA | NA | NA |  | NA |
|  | cg08060987 | downstream | NA |  |  | NA |  |
|  | cg17250225 | downstream | NA | NA | NA |  | NA |
|  | cg24962576 | downstream | NA |  |  |  |  |
|  | N° of altered CpGs / interrogated CpGs | | 2/2 | 1/9 | 2/9 | 0/11 | 0/9 |
| **chr5:140865268-140865524 Nearest TSS gene: *PCDHG* cluster** | cg26890354 | upstream | NA |  |  |  |  |
|  | cg06428620 | upstream | NA |  |  | NA |  |
|  | cg12648074 | upstream | NA |  |  |  |  |
|  | cg15949044 | upstream | NA |  |  | NA |  |
|  | cg02022733 | upstream | NA |  |  |  |  |
|  | cg03640756 | upstream | NA |  |  | NA |  |
|  | cg15361590 | upstream |  |  |  | NA |  |
|  | cg23445461 | upstream | NA |  |  |  |  |
|  | cg12145907 | upstream |  |  |  | NA |  |
|  | cg11830096 | within | NA |  |  |  |  |
|  | cg00163472 | downstream | NA | NA | NA |  | NA |
|  | cg17115609 | downstream | NA | NA | NA |  | NA |
|  | cg15489171 | downstream | NA | NA | NA |  | NA |
|  | cg00943245 | downstream |  |  |  |  |  |
|  | N° of altered CpGs / interrogated CpGs | | 1/3 | 2/11 | 8/11 | 0/9 | 0/11 |
| **chr11:58343145-58343421 Nearest TSS gene: LPXN** | cg23907504 | upstream | NA | NA | NA |  | NA |
|  | cg20654468 | upstream |  |  |  |  |  |
|  | cg02044895 | within | NA |  |  |  |  |
|  | cg23641267 | upstream |  |  |  |  |  |
|  | cg12891342 | upstream | NA |  |  |  |  |
|  | cg26534696 | upstream | NA | NA | NA |  | NA |
|  | cg08823864 | upstream | NA | NA | NA |  | NA |
|  | cg00501111 | upstream | NA |  |  |  |  |
|  | cg09383596 | upstream | NA |  |  | NA |  |
|  | cg23559222 | upstream | NA |  |  | NA |  |
|  | N° of altered CpGs / interrogated CpGs | | 1/2 | 2/7 | 1/7 | 1/8 | 0/7 |
| **chr11:58344505-58344915 Nearest TSS gene: *LPXN*** | cg20654468 | upstream |  |  |  |  |  |
|  | cg02044895 | upstream | NA |  |  |  |  |
|  | cg23641267 | upstream |  |  |  |  |  |
|  | cg12891342 | upstream | NA |  |  |  |  |
|  | cg26534696 | upstream | NA | NA | NA |  | NA |
|  | cg08823864 | within | NA | NA | NA |  | NA |
|  | cg00501111 | downstream | NA |  |  |  |  |
|  | cg09383596 | downstream | NA |  |  | NA |  |
|  | cg23559222 | downstream | NA |  |  | NA |  |
|  | cg05013250 | downstream | NA |  |  |  |  |
|  | cg19963142 | downstream | NA |  |  |  |  |
|  | cg26151079 | downstream | NA |  |  | NA |  |
|  | cg04570322 | downstream | NA |  |  | NA |  |
|  | cg24684739 | downstream | NA |  |  |  |  |
|  | cg20957095 | downstream | NA |  |  | NA |  |
|  | cg16503683 | downstream | NA |  |  | NA |  |
|  | cg04050763 | downstream | NA |  |  |  |  |
|  | cg06677781 | downstream | NA |  |  |  |  |
|  | cg16725583 | downstream | NA |  |  |  |  |
|  | cg04829746 | downstream | NA |  |  | NA |  |
|  | cg24899209 | downstream | NA |  |  | NA | NA |
|  | cg02250680 | downstream | NA |  |  |  |  |
|  | N° of altered CpGs / interrogated CpGs | | 1/2 | 2/20 | 1/20 | 1/14 | 0/19 |
| **chr16:70679121-70679531 Nearest TSS gene: *IL34*** | cg08281777 | within | NA |  |  |  |  |
|  | cg04917208 | downstream | NA |  |  |  |  |
|  | cg01447350 | downstream | NA |  |  | NA |  |
|  | cg04509100 | downstream | NA |  |  | NA | NA |
|  | cg01782798 | downstream | NA |  |  |  |  |
|  | cg12347740 | downstream |  |  |  |  |  |
|  | cg26831220 | downstream | NA |  |  |  |  |
|  | cg04164048 | downstream | NA |  |  |  |  |
|  | cg04012053 | downstream | NA |  |  |  |  |
|  | cg08402568 | downstream |  |  |  |  |  |
|  | N° of altered CpGs / interrogated CpGs | | 1/2 | 7/10 | 1/10 | 0/8 | 0/9 |

Note. NA: not available, red: hypermethylation and blue: hypomethylation.

**Supplementary Fig. 8**

**
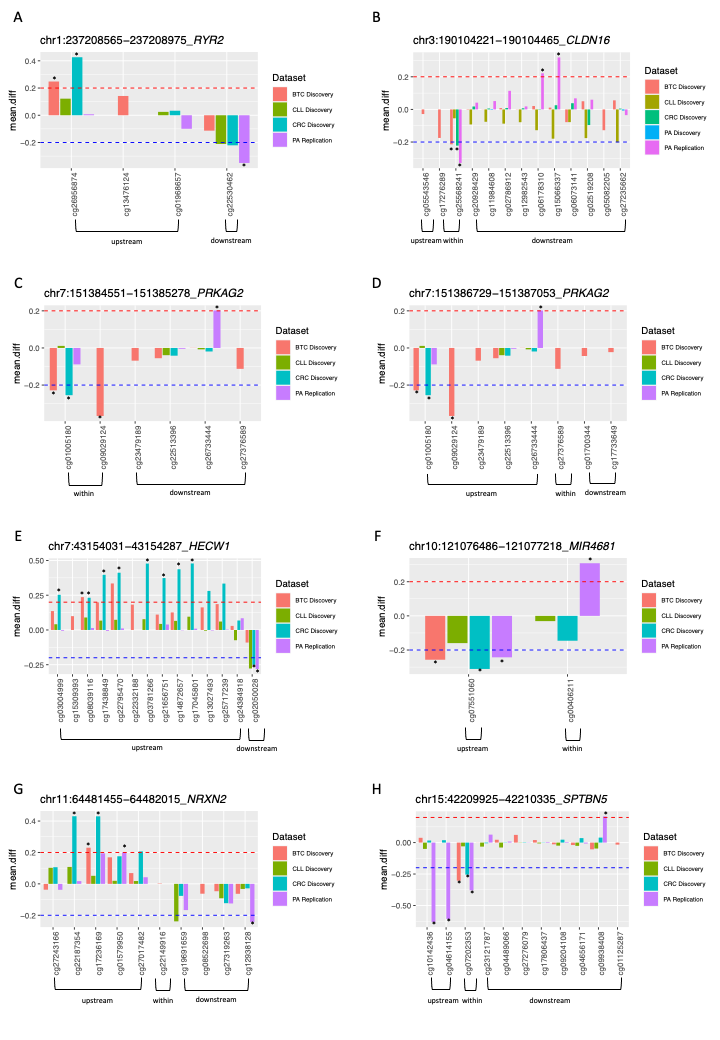
**

**Supplementary Fig. 8**. Additional NRSE alterations shared between PA Replication and the other analysed datasets, BTC, CLL and CRC Discovery. Bar plots representing the differential methylation value between tumour and normal samples of the selected NRSE regions associated with the following genes: *RYR2* (A), *CLDN16* (B), *PRKAG2* (C and D), *HECW1* (E), *MIR4681* (F), *NRXN2* (G) and *SPTBN5* (H). Asterisks indicate significant and validated CpGs.

**Supplementary Table 2**. Eight altered NRSE regions, including all interrogated CpGs, shared between PA Replication and the other analysed datasets, BTC, CLL and CRC Discovery.

| **NRSE** | **CpGs interrogated** | **Relation to NRSE** | **PA Replication** | **CRC Discovery** | **BTC Discovery** | **CLL Discovery** |
| --- | --- | --- | --- | --- | --- | --- |
| **chr1:91189399-91189655 Nearest TSS gene: *BARHL2*** | cg16058493 | upstream |  |  | NA |  |
|  | cg15469272 | upstream |  |  | NA |  |
|  | cg02595750 | within |  |  | NA |  |
|  | cg25416818 | within | NA | NA |  | NA |
|  | cg21542223 | downstream |  |  |  |  |
|  | cg26568372 | downstream |  |  | NA |  |
|  | cg17480076 | downstream |  |  | NA |  |
|  | cg05039548 | downstream |  |  |  |  |
|  | cg20634266 | downstream |  |  |  |  |
|  | cg19414591 | downstream |  |  |  |  |
|  | cg15452970 | downstream |  |  |  |  |
|  | cg24453699 | downstream |  |  | NA |  |
|  | cg20463526 | downstream |  |  |  |  |
|  | cg26072058 | downstream |  |  | NA |  |
|  | cg08062812 | downstream |  |  |  |  |
|  | cg15992284 | downstream |  |  | NA |  |
|  | cg13456504 | downstream |  |  | NA |  |
|  | N° of altered CpGs / interrogated CpGs | | 1/16 | 4/16 | 5/8 | 0/16 |
| **chr6:31026052-31026462 Nearest TSS gene: *HCG22*** | cg08651007 | upstream |  |  |  |  |
|  | cg11039913 | upstream |  |  |  |  |
|  | cg00583046 | upstream |  |  |  |  |
|  | cg27644095 | upstream |  |  |  |  |
|  | cg15520752 | upstream | NA | NA |  | NA |
|  | cg13525397 | upstream |  |  |  |  |
|  | cg24764810 | upstream |  |  |  |  |
|  | cg14343414 | upstream |  |  |  |  |
|  | cg08448622 | within |  |  |  |  |
|  | cg19195712 | within |  |  | NA |  |
|  | cg07717512 | within |  |  | NA |  |
|  | cg22880122 | downstream |  |  |  |  |
|  | N° of altered CpGs / interrogated CpGs | | 3/11 | 4/11 | 1/10 | 0/11 |
| **chr7:8476565-8476895 Nearest TSS gene: *NXPH1*** | cg19469357 | upstream |  |  | NA |  |
|  | cg02124383 | upstream |  |  |  |  |
|  | cg13578408 | upstream |  |  |  |  |
|  | cg00399951 | upstream |  |  |  |  |
|  | cg06056929 | upstream |  |  | NA |  |
|  | cg17264513 | upstream |  |  |  |  |
|  | cg00288806 | upstream |  |  | NA |  |
|  | cg25708069 | within |  |  | NA |  |
|  | cg17278735 | within |  |  |  |  |
|  | cg20378002 | downstream |  |  | NA |  |
|  | cg06981279 | downstream |  |  |  |  |
|  | cg03836586 | downstream |  |  | NA |  |
|  | N° of altered CpGs / interrogated CpGs | | 1/12 | 1/12 | 1/6 | 0/12 |
| **chr14:57262782-57263038 Nearest TSS gene: *OTX2*** | cg13080734 | upstream | NA | NA |  | NA |
|  | cg05413872 | upstream |  |  | NA |  |
|  | cg06633061 | upstream |  |  | NA |  |
|  | cg12710899 | upstream |  |  | NA |  |
|  | cg00107772 | upstream |  |  | NA |  |
|  | cg08235161 | upstream |  |  | NA |  |
|  | cg21106407 | upstream |  |  | NA |  |
|  | cg04508739 | downstream |  |  |  |  |
|  | cg18389933 | downstream |  |  |  |  |
|  | cg19787727 | downstream |  |  |  |  |
|  | cg02237629 | downstream |  |  |  |  |
|  | cg19871940 | downstream |  |  |  |  |
|  | cg02298116 | downstream |  |  | NA |  |
|  | cg24292703 | downstream |  |  | NA |  |
|  | cg12853633 | downstream |  |  |  |  |
|  | N° of altered CpGs / interrogated CpGs | | 3/14 | 9/14 | 3/7 | 0/14 |
| **chr17:38470828-38471318 Nearest TSS gene: *RARA*** | cg25362050 | within |  |  |  |  |
|  | cg00442282 | within |  |  |  |  |
|  | cg21238609 | downstream |  |  |  |  |
|  | cg19458020 | downstream |  |  |  |  |
|  | cg15803221 | downstream |  |  |  |  |
|  | cg02898721 | downstream |  |  |  |  |
|  | N° of altered CpGs / interrogated CpGs | | 3/6 | 1/6 | 1/6 | 0/6 |
| **chr17:38471678-38472235 Nearest TSS gene: *RARA*** | cg25362050 | upstream |  |  |  |  |
|  | cg00442282 | upstream |  |  |  |  |
|  | cg21238609 | downstream |  |  |  |  |
|  | cg19458020 | downstream |  |  |  |  |
|  | cg15803221 | downstream |  |  |  |  |
|  | cg02898721 | downstream |  |  |  |  |
|  | cg08005460 | downstream |  |  | NA |  |
|  | cg11501059 | downstream | NA | NA |  | NA |
|  | N° of altered CpGs / interrogated CpGs | | 3/7 | 1/7 | 1/7 | 0/7 |
| **chr19:44205502-44205992 Nearest TSS gene: *IRGC*** | cg24399430 | upstream |  |  |  |  |
|  | cg08669447 | upstream |  |  |  |  |
|  | cg09489306 | upstream |  |  |  |  |
|  | cg00535514 | within |  |  | NA |  |
|  | cg12297819 | downstream |  |  |  |  |
|  | N° of altered CpGs / interrogated CpGs | | 1/5 | 1/5 | 2/4 | 0/5 |
| **chr19:46714429-46714919 Nearest TSS gene: *LOC93429*** | cg02088550 | upstream | NA | NA |  | NA |
|  | cg21100328 | upstream |  |  |  |  |
|  | cg14330675 | upstream |  |  |  |  |
|  | cg21629821 | upstream |  |  | NA |  |
|  | cg00145150 | upstream |  |  | NA |  |
|  | cg24473594 | within |  |  |  |  |
|  | cg02719380 | downstream | NA | NA |  | NA |
|  | N° of altered CpGs / interrogated CpGs | | 1/5 | 1/5 | 1/5 | 0/5 |

Note. NA: not available, red: hypermethylation and blue: hypomethylation.

**Supplementary Table 3**. REST interactors and other factors binding in the selected NRSE regions in H1-hESC cell line.

| **NRSE** | **Transcription factor symbol** | **Transcription factor description** | **Chromosome** | **Start** | **End** | **Score** |
| --- | --- | --- | --- | --- | --- | --- |
| chr1:91189399-91189655 Nearest TSS gene: *BARHL2* | ASH2L | ASH2 Like, Histone Lysine Methyltransferase Complex Subunit | chr1 | 91188987 | 91189767 | 492 |
|  | CBX8 | Chromobox 8 | chr1 | 91191599 | 91192426 | 1000 |
|  | CHD7 | Chromodomain Helicase DNA Binding Protein 7 | chr1 | 91186886 | 91187462 | 565 |
|  | CTCF | CCCTC-Binding Factor | chr1 | 91189080 | 91190015 | 1000 |
|  |  |  | chr1 | 91186488 | 91187747 | 1000 |
|  | EGR1 | Early Growth Response 1 | chr1 | 91189335 | 91189605 | 397 |
|  | KDM4A | Lysine Demethylase 4A | chr1 | 91190173 | 91191791 | 348 |
|  | KDM5A | Lysine Demethylase 5A | chr1 | 91189223 | 91189647 | 189 |
|  | NANOG | Nanog Homeobox | chr1 | 91191195 | 91191445 | 340 |
|  | RAD21 | RAD21 Cohesin Complex Component | chr1 | 91189363 | 91189573 | 1000 |
|  |  |  | chr1 | 91191158 | 91191444 | 210 |
|  | RBBP5 | RB Binding Protein 5, Histone Lysine Methyltransferase Complex Subunit | chr1 | 91190628 | 91191272 | 625 |
|  | RNF2 | Ring Finger Protein 2 | chr1 | 91188317 | 91189295 | 528 |
|  |  |  | chr1 | 91189395 | 91189783 | 134 |
|  |  |  | chr1 | 91190445 | 91193163 | 1000 |
|  | RXRA | Retinoid X Receptor Alpha | chr1 | 91189342 | 91189558 | 478 |
|  | SIN3A | SIN3 Transcription Regulator Family Member A | chr1 | 91189155 | 91189675 | 676 |
|  | SP1 | Sp1 Transcription Factor | chr1 | 91189291 | 91189846 | 284 |
|  | SUZ12 | SUZ12 Polycomb Repressive Complex 2 Subunit | chr1 | 91188335 | 91193773 | 1000 |
|  | TCF12 | Transcription Factor 12 | chr1 | 91189322 | 91189582 | 478 |
|  | YY1 | YY1 Transcription Factor | chr1 | 91189335 | 91189671 | 264 |
|  | ZNF143 | Zinc Finger Protein 143 | chr1 | 91189314 | 91189604 | 429 |
|  | HDAC2* | Histone Deacetylase 2 | chr1 | 91163185 | 91204211 | 344 |
|  |  |  | chr1 | 91189416 | 91189529 | 1000 |
|  |  |  | chr1 | 91190055 | 91190174 | 971 |
|  |  |  | chr1 | 91190472 | 91190663 | 898 |
|  |  |  | chr1 | 91191092 | 91191359 | 918 |
|  | HDAC6* | Histone Deacetylase 6 | chr1 | 91109620 | 91375160 | 256 |
| chr14:57262782-57263038 Nearest TSS gene: *OTX2* | ASH2L | ASH2 Like, Histone Lysine Methyltransferase Complex Subunit | chr14 | 57260862 | 57261642 | 401 |
|  |  |  | chr14 | 57263769 | 57264549 | 285 |
|  | CHD7 | Chromodomain Helicase DNA Binding Protein 7 | chr14 | 57262926 | 57263502 | 382 |
|  | EGR1 | Early Growth Response 1 | chr14 | 57260888 | 57261158 | 120 |
|  | FOSL1 | FOS Like 1, AP-1 Transcription Factor Subunit | chr14 | 57260918 | 57261154 | 207 |
|  | GABPA | GA Binding Protein Transcription Factor Subunit Alpha | chr14 | 57260863 | 57261179 | 1000 |
|  |  |  | chr14 | 57264064 | 57264408 | 185 |
|  | HDAC2 | Histone Deacetylase 2 | chr14 | 57260923 | 57261782 | 681 |
|  |  |  | chr14 | 57264564 | 57264820 | 554 |
|  | JUN | Jun Proto-Oncogene, AP-1 Transcription Factor Subunit | chr14 | 57262816 | 57263060 | 356 |
|  | JUND | JunD Proto-Oncogene, AP-1 Transcription Factor Subunit | chr14 | 57260926 | 57261170 | 288 |
|  | JUND |  | chr14 | 57262924 | 57263240 | 1000 |
|  | KDM4A | Lysine Demethylase 4A | chr14 | 57260926 | 57262165 | 601 |
|  |  |  | chr14 | 57263947 | 57265054 | 650 |
|  | PHF8 | PHD Finger Protein 8 | chr14 | 57260957 | 57261861 | 433 |
|  | POLR2A | RNA Polymerase II Subunit A | chr14 | 57260803 | 57261447 | 409 |
|  | RAD21 | RAD21 Cohesin Complex Component | chr14 | 57261001 | 57261245 | 55 |
|  | RBBP5 | RB Binding Protein 5, Histone Lysine Methyltransferase Complex Subunit | chr14 | 57261026 | 57261885 | 783 |
|  |  |  | chr14 | 57264758 | 57265781 | 705 |
|  | RNF2 | Ring Finger Protein 2 | chr14 | 57260908 | 57262176 | 536 |
|  |  |  | chr14 | 57264398 | 57265397 | 209 |
|  | SAP30 | Sin3A Associated Protein 30 | chr14 | 57264049 | 57264519 | 589 |
|  | SIN3A | SIN3 Transcription Regulator Family Member A | chr14 | 57260785 | 57261305 | 439 |
|  |  |  | chr14 | 57264132 | 57264854 | 563 |
|  | SP1 | Sp1 Transcription Factor | chr14 | 57263090 | 57263410 | 192 |
|  | SRF | Serum Response Factor | chr14 | 57260931 | 57261161 | 285 |
|  | SUZ12 | SUZ12 Polycomb Repressive Complex 2 Subunit | chr14 | 57260867 | 57262952 | 539 |
|  |  |  | chr14 | 57263843 | 57265620 | 595 |
|  | TAF1 | TATA-Box Binding Protein Associated Factor 1 | chr14 | 57260875 | 57261130 | 449 |
|  |  |  | chr14 | 57261553 | 57262043 | 152 |
|  | TBP | TATA-Box Binding Protein | chr14 | 57260828 | 57261208 | 505 |
|  |  |  | chr14 | 57261750 | 57262130 | 139 |
|  | YY1 | YY1 Transcription Factor | chr14 | 57260806 | 57261142 | 280 |
|  | ZNF143 | Zinc Finger Protein 143 | chr14 | 57260898 | 57261188 | 324 |
|  |  |  | chr14 | 57261779 | 57262069 | 179 |
|  | HDAC2* | Histone Deacetylase 2 | chr14 | 57260044 | 57266941 | 596 |
|  | HDAC6* | Histone Deacetylase 6 | chr14 | 57254534 | 57293455 | 391 |
| chr5:140865268-140865524 Nearest TSS gene: *PCDHG* cluster | ASH2L | ASH2 Like, Histone Lysine Methyltransferase Complex Subunit | chr5 | 140863441 | 140865166 | 673 |
|  | CTCF | CCCTC-Binding Factor | chr5 | 140864401 | 140864617 | 132 |
|  | HDAC2 | Histone Deacetylase 2 | chr5 | 140864386 | 140865042 | 904 |
|  | JUND | JunD Proto-Oncogene, AP-1 Transcription Factor Subunit | chr5 | 140864450 | 140864694 | 395 |
|  | NANOG | Nanog Homeobox | chr5 | 140864285 | 140864737 | 122 |
|  | PHF8 | PHD Finger Protein 8 | chr5 | 140864141 | 140864705 | 136 |
|  | POLR2A | RNA Polymerase II Subunit A | chr5 | 140864308 | 140864952 | 401 |
|  | RAD21 | RAD21 Cohesin Complex Component | chr5 | 140864408 | 140864912 | 126 |
|  | RBBP5 | RB Binding Protein 5, Histone Lysine Methyltransferase Complex Subunit | chr5 | 140863986 | 140864901 | 561 |
|  | RFX5 | Regulatory Factor X5 | chr5 | 140864047 | 140864667 | 1000 |
|  | SAP30 | Sin3A Associated Protein 30 | chr5 | 140864496 | 140865184 | 471 |
|  | SIN3A | SIN3 Transcription Regulator Family Member A | chr5 | 140864183 | 140864881 | 547 |
|  | SP1 | Sp1 Transcription Factor | chr5 | 140863407 | 140863727 | 288 |
|  |  |  | chr5 | 140864380 | 140864700 | 319 |
|  | TAF1 | TATA-Box Binding Protein Associated Factor 1 | chr5 | 140864360 | 140864850 | 260 |
|  | HDAC2* | Histone Deacetylase 2 | chr5 | 140863994 | 140865462 | 615 |
|  | HDAC6* | Histone Deacetylase 6 | chr5 | 140665501 | 141439702 | 284 |

**Supplementary Table 4**. Histone modifications in the selected NRSE regions in H1-hESC cell line.

|  |  |  | **chr1:91189399-91189655**  **Nearest TSS gene: *BARHL2*** | | | | **chr14:57262782-57263038 Nearest TSS gene: *OTX2*** | | | | **chr5:140865268-140865524 Nearest TSS gene: *PCDHG* cluster** | | | |
| --- | --- | --- | --- | --- | --- | --- | --- | --- | --- | --- | --- | --- | --- | --- |
| **Histone modification** | **Genomic distribution** | **Related function** | **Chr** | **Start** | **End** | **Score** | **Chr** | **Start** | **End** | **Score** | **Chr** | **Start** | **End** | **Score** |
| H3K27ac | active enhancers, promoters | transcriptional activation | -- | -- | -- | -- | chr14 | 57253557 | 57285156 | 341 | chr5 | 140854108 | 140879610 | 283 |
|  |  |  |  |  |  |  |  |  |  |  | chr5 | 140863746 | 140863956 | 833 |
| H3K27m3 | inactive promoters | transcriptional repression | chr1 | 91160227 | 91209545 | 1000 | chr14 | 57248640 | 57296485 | 676 | chr5 | 140863849 | 140868561 | 583 |
| H3K36m3 | gene bodies | transcriptional activation, RNA splicing | -- | -- | -- | -- | -- | -- | -- | -- | chr5 | 140856961 | 140877110 | 284 |
| H3K4m1 | poised enhancers, promoters | enhancer priming, transcriptional activation | chr1 | 91162682 | 91204446 | 424 | chr14 | 57251136 | 57294420 | 480 | chr5 | 140851263 | 140880739 | 414 |
|  |  |  |  |  |  |  |  |  |  |  | chr5 | 140862219 | 140866332 | 566 |
| H3K4m2 |  | transcriptional activation | chr1 | 91188060 | 91197553 | 744 | chr14 | 57251140 | 57294521 | 708 | chr5 | 140862351 | 140866442 | 954 |
| H3K4m3 | active promoters | transcriptional activation | chr1 | 91189142 | 91189488 | 580 | chr14 | 57251236 | 57294168 | 457 | chr5 | 140862756 | 140866276 | 565 |
|  |  |  | chr1 | 91190050 | 91193188 | 429 |  |  |  |  |  |  |  |  |
| H3K79m2 |  | transcriptional activation | -- | -- | -- | -- | -- | -- | -- | -- | chr5 | 140853827 | 140876907 | 282 |
| H3K9ac | active promoter | transcriptional activation | chr1 | 91189657 | 91190705 | 368 | chr14 | 57251171 | 57290893 | 392 | chr5 | 140846025 | 140881907 | 306 |
|  |  |  |  |  |  |  | chr14 | 57260676 | 57262509 | 509 |  |  |  |  |
|  |  |  | chr1 | 91191015 | 91192442 | 347 | chr14 | 57263889 | 57264020 | 884 |  |  |  |  |
|  |  |  |  |  |  |  | chr14 | 57264764 | 57264880 | 818 |  |  |  |  |
| H3K9m3 | heterochromatin | transcriptional repression, heterochromatin formation | chr1 | 91184466 | 91194895 | 286 | -- | -- | -- | -- | -- | -- | -- | -- |
| H4K20m1 |  | transcriptional repression | chr1 | 91161569 | 91209017 | 413 | chr14 | 57256764 | 57292959 | 349 | chr5 | 140847389 | 140880920 | 325 |

Note. Genomic distribution and related function were obtained from [1,2].

1. Steinbach N. PTEN affects gene expression and histone modifications and plays a role in the regulation of transcription [Internet]. Columbia University; 2017 [cited 2023 May 4]. Available from: https://doi.org/10.7916/D8GH9W9R

2. Nag M, De Paris K, E Fogle J. Epigenetic Modulation of CD8^+^ T Cell Function in Lentivirus Infections: A Review. Viruses. 2018;10:227.
